# Elucidating the Molecular Mechanisms of *Vitex negundo* L. in Polycystic Ovary Syndrome via Metabolomics‐Driven Network Pharmacology

**DOI:** 10.1002/bmc.70592

**Published:** 2026-08-10

**Authors:** Nishmitha R. Poojary, Neeharika Narisepalli Venkatasai, Kannath U. Sanjay, Supraja M. Kodanch, Padmalatha S. Rai

**Affiliations:** ^1^ Department of Biotechnology, Manipal School of Life Sciences Manipal Academy of Higher Education Manipal Karnataka India

**Keywords:** molecular docking, network pharmacology, polycystic ovary syndrome, untargeted metabolomics, *Vitex negundo*

## Abstract

Polycystic ovary syndrome (PCOS) is a hormonal disorder marked by irregular menstrual cycles, elevated androgen levels, ovarian cysts, hirsutism, acne and other symptoms. While conventional medications such as Metformin and Spironolactone are commonly prescribed, they are often associated with undesirable side effects. As a result, there is growing interest in alternative treatments, particularly those involving medicinal plants. *Vitex negundo* L., a member of the Lamiaceae family, has demonstrated promising therapeutic effects against PCOS. However, its precise molecular mechanism of action remains unclear. To explore this, we conducted an untargeted metabolomics analysis using UPLC‐MS/MS to identify bioactive compounds, followed by network pharmacology to elucidate potential mechanisms. Metabolite fingerprinting revealed 186 metabolites, among which 122 were identified as secondary metabolites. Network pharmacology analysis uncovered 910 potential targets associated with the identified compounds and 297 known PCOS‐related disease targets, with 50 overlapping targets between the two datasets. Key hub targets identified included P53, ESR1, AKT1, STAT3, CTNNB1, ERBB2, BCL2, EGFR, MTOR and IL6. Furthermore, molecular docking highlighted several bioactive constituents—syringin, 4‐(3,4‐dihydroxyphenyl)‐6,7‐dihydroxynaphthalene‐2‐carboxylic acid, and Isovitexin—as potential lead compounds for PCOS treatment. Further evaluation of lead compounds can be conducted through in vitro and in vivo studies.

## 1 Introduction

Polycystic ovary syndrome (PCOS) is a hormonal condition that affects approximately 4%–20% of women globally (Teede et al. 2024). The features of PCOS include irregular menstrual cycles, high levels of androgen, hirsutism, acne, anovulation and alopecia (Rashid et al. 2022). The root cause of PCOS is unknown, but it can be influenced by lifestyle, environmental and genetic factors (Kar et al. 2024). PCOS results in inflammation of the ovaries, which lowers the egg quality and causes irregular ovulation, leading to infertility. The diagnosis of PCOS is based on key parameters, such as hyperandrogenism, anovulation and polycystic ovaries. According to the Rotterdam criteria, two of these three key parameters should be present for the diagnosis of PCOS (Christ and Cedars 2023). The purpose of PCOS treatment is to maintain BMI, androgen levels and normalise ovulatory cycles. Metformin, Spironolactone and birth control pills are the standard medications that are used to treat PCOS. Existing medication has many side effects and only gives short‐term relief from the symptoms (Kar et al. 2024). These synthetic drugs are known to cause adverse side effects such as nausea, cardiovascular diseases, weight gain and lactic acidosis (Rashid et al. 2022). Therefore, as an alternative to synthetic drugs, phytotherapy is used as it contains active molecules and has minimal side effects (Sharma and Yadav 2022).

*Vitex negundo* L., also known as “Nirgundi,” is a shrub belonging to the Lamiaceae family known for its remarkable medicinal benefits (Sharma et al. 2023). It often grows in moist areas, up to 1500 m above sea level, in the Mediterranean and Central Asian. This plant is identified by its four‐angled branches, lanceolate leaves consisting of 3–5 leaflets and small bluish‐purple flowers. *V. negundo* contains a wide range of bioactive compounds, including flavonoids, iridoids, lignans and terpenoids. These metabolites play an important role in regulating signalling pathways, cell cycle, apoptosis, PCOS and the menstrual cycle (Gill et al. 2018). *V. negundo* is mainly utilised due to its anti‐inflammatory, antitumour and antimicrobial properties. It plays an essential role in controlling diabetes and improving reproductive health (Kakadia et al. 2019). It was found that the fruit extract of *V. negundo* showed enhanced aromatase activity and reduced testosterone levels. Parts of this plant, including leaves, flowers, roots and germinating seeds, have medicinal benefits (Kar et al. 2024).

Recent drug discovery strategies often follow a single‐drug, single‐target, single‐disease approach, which is inefficient in clinical treatments. However, single‐target approaches may help treat gene disorders associated with a single gene but are not effective for complex diseases involving multiple genes. Hence, developing novel drugs that target multiple sites for complex diseases is needed. In this regard, network pharmacology has emerged as one of the most advanced fields in drug development and discovery, integrating systems medicine with information science. To understand the therapeutic effects of traditional medicine, protein‐compound/disease–gene networks are utilised. It is currently employed in three different fields: conventional medicine, pharmacology and drug research. In the field of traditional medicine, it helps in understanding the mechanism of drug action and acts as a biomarker in quality control. It also helps to develop novel drugs from natural products and understand their mechanisms, side effects and drug interactions (Noor et al. 2022).

Metabolomics can be broadly categorised into two types based on data acquisition: targeted and untargeted. Untargeted metabolomics involves a comprehensive analysis of the entire metabolome, while targeted metabolomics focuses on a specific set of selected metabolites from one or more pathways. These small molecules, collectively known as the metabolome, are integral to numerous biochemical pathways within living organisms. Compared to other “omics” approaches, such as proteomics, genomics and transcriptomics, which examine upstream molecular changes, metabolomics provides a direct, holistic view of metabolic alterations, thereby offering insights into biochemical, physiological and genetic changes (Guo et al. 2022). In plant research, metabolomics plays a crucial role in identifying low‐molecular weight compounds, as plant metabolites are significantly influenced by both genetic makeup and environmental conditions (Waris et al. 2022). Consequently, metabolomic studies are essential for detecting and analysing plant metabolites based on factors such as growth stage, plant part, strain and extraction method. Metabolite fingerprinting thus enables the identification of bioactive compounds at the molecular level (Huang et al. 2024). In this context, the present study aims to perform metabolomic profiling of *V. negundo* to identify its lead bioactive compounds and elucidate the mechanisms through which these compounds may exert therapeutic effects against PCOS.

## 2 Methods

### 2.1 Sample Collection and DNA Barcoding

*V. negundo* was collected from Udupi, Karnataka, India. Subsequently, the herbarium specimen was prepared, and a specific accession number was assigned. The germplasm of this plant was maintained in the greenhouse at the Manipal School of Life Sciences (MSLS), MAHE, Manipal. A modified CTAB protocol was used to isolate the DNA (Tiwari et al. 2012), and PCR was subsequently performed using a Veriti thermal cycler (Applied Biosystems, USA). The PCR master mixture consists of 10 μL of Milli‐Q, 10 μL of PCR mix, 1 μL of forward primer, 1 μL of reverse primer and 1 μL of genomic DNA. The PCR product was checked, and results were observed in a 1.2% agarose gel; later, it was sequenced using a Genetic Analyser. The PCR reaction conditions are provided in Table S1. The DNA barcode sequence was submitted to NCBI GenBank, and an accession number was obtained.

### 2.2 Metabolite Fingerprinting

The leaves of *V. negundo* were sterilised and ground with liquid nitrogen. To extract the metabolites, ice‐cold acidified methanolic extraction was performed (De Vos et al. 2007). Later, the sample was reconstituted and filtered using a 0.22‐μm PTFE filter. The samples were injected in triplicate to assess analytical reproducibility. UPLC‐MS/MS analysis was carried out using a QExactive Orbitrap high‐resolution mass spectrometer coupled with a Vanquish UPLC system equipped with a Kinetex reverse‐phased C18 column. The UPLC conditions and gradient run programme were chosen as previously described (Vinay et al. 2025). A QExactive Orbitrap with an electrospray ionisation (ESI) source was used for mass spectrometric analysis. It operated at a resolution of 140,000 for both full scan (MS1) and data‐dependent MS/MS acquisition. The device switches between positive and negative ionisation modes. Stepped normalised collision energies of 30, 40 and 100 were used for data‐dependent MS/MS. For both MS1 and MS/MS scans, metabolite detection was performed within a mass‐to‐charge (m/z) range of 200–2000. Thermo Xcalibur 2.0 software (Thermo Fisher Scientific, USA) was used to obtain raw data.

### 2.3 Data Analysis

The raw data obtained from the UPLC‐MS/MS are converted to the mzML format using the ProteoWizard msConvert GUI software. The mzML file was then exported to MS‐DIAL software 4.9 (Tsugawa et al. 2015) to process mzML files in centroid mode for both positive and negative ionisation. The parameters for MS‐DIAL included an MS1 tolerance of 0.01 Da and an MS2 tolerance of 0.025 Da. The minimum peak height was set to 30,000, the mass slice width was 0.1 Da and the sigma window value was 1. The MS/MS abundance cutoff was set at 10, and the retention time tolerance was 0.05 min. Adduct consideration for metabolite annotation include [M + H]+, [M + Na]+, [M + NH4]+, [M + K]+, [2 M + NH4]+, [2 M + H]+, [2 M + Na]+ and [2 M + K]+ for positive mode and [M‐H]‐, [2 M‐H]‐, [M + FA‐H]‐ and [2 M + FA‐H]‐ for negative mode. Tentative metabolite identification was performed by matching MS/MS spectra from positive and negative ion modes against public libraries, including MassBank, ReSpect, GNPS and Fiehn/Vaniya Natural Product. A PPM error less than 15 were considered for final filtering for metabolite annotation. After initial feature annotation, a manual curation was conducted to ensure the biological relevance. During this process, ambiguous, misannotated and nonendogenous annotations were removed. PubChem database was used to get the SMILES and the PubChem CID of the metabolites (Jakatimath et al. 2024; Gunasekaran and Kalpana 2026).

### 2.4 Target Prediction

The SMILES of metabolites were provided as input to retrieve compound targets from SwissTargetPrediction. Metabolite targets with a probability of greater than 0.1 were selected. PCOS disease‐related targets were retrieved from GeneCards (relevance score > 50) and DisGeNET (score > 0.75) by giving the keyword “polycystic ovary syndrome”. The Venny 2.1 tool was used to perform intersectional analysis to identify common targets.

### 2.5 Protein–Protein Interaction (PPI) Network

Common targets were provided as input to STRINGdb in Cytoscape (Version 3.10.3) (Szklarczyk et al. 2015), and a confidence score of 0.9 (high confidence) was used to generate the PPI network. The PPI network was used (Shannon et al. 2003) to identify densely connected protein clusters. The hub targets were determined using the cyto‐hubba plugin (Chin et al. 2014), and the top 10 hub targets were identified based on degree centrality.

### 2.6 Functional Enrichment

Gene ontology (GO) and KEGG analysis were performed using ShinyGO (Ge et al. 2020) to investigate the biological processes, cellular components, molecular functions and associated pathways of the hub targets.

### 2.7 Drug Likeness Prediction and Molecular Docking

SMILES of secondary metabolites in *V. negundo* were retrieved from Pubchem and given as an input for the Ligprep tool in Schrodinger Suite to prepare the ligands. All the prepared ligands were used to predict drug‐like properties using Qikprop. Lipinski's rule of five was considered to determine the drug‐like properties. Ligands exhibiting drug‐like properties were considered for molecular docking studies. Protein preparation was performed using the Protein Preparation Wizard. The site map module was used to predict the potential binding sites in the target protein. The site with the top score was selected for grid generation using the receptor grid generation module. Molecular docking was performed using the Glide module of the Schrodinger suite.

## 3 Results

### 3.1 Sample Collection and Molecular Authentication

The *V. negundo* samples were collected from Udyavara, Udupi, Karnataka, India. The herbarium (Figure S1) was prepared and stored at the MSLS, MAHE, Manipal and molecular authentication was performed using DNA barcoding with the *nrITS* marker (Figures S2 and S3). The DNA barcode sequence was submitted to NCBI GenBank (accession number: PV710460).

### 3.2 Metabolite Fingerprinting

**FIGURE 1:**
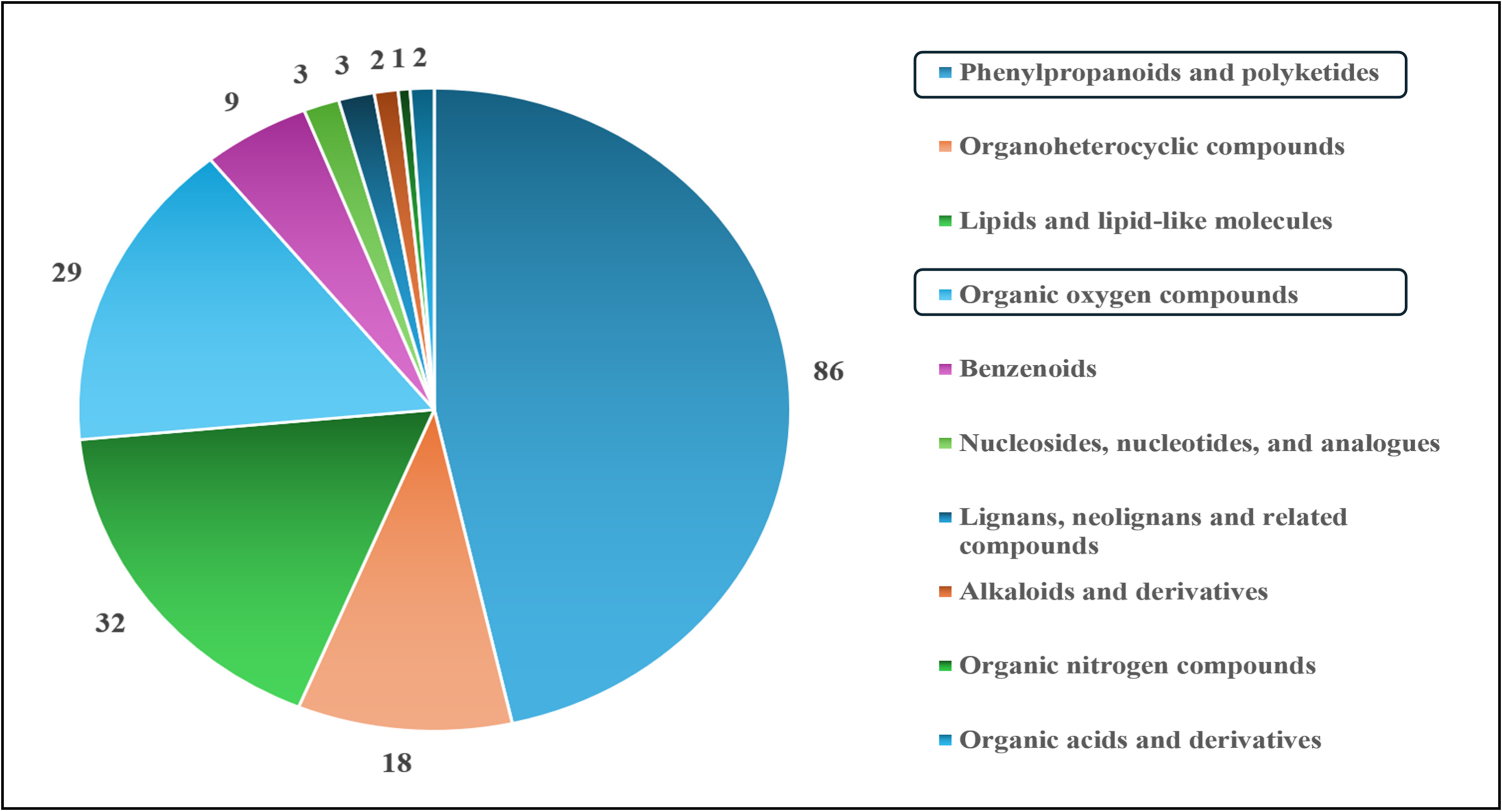
Pie chart representing the classification of identified metabolites.

A total of 186 putative metabolites were shortlisted from *V. negundo* using UPLC‐MS/MS. The compound names, experimental m/z values, retention time, molecular formulas and part‐per‐million errors (< 10 ppm) are presented in Table S2. Metabolites were classified based on their compound superclass using the ClassyFire tool (Djoumbou Feunang et al. 2016). Most of the metabolites belong to the superclass phenylpropanoids and polyketides (46%), lipids and lipid‐like molecules (17%), organic oxygen compounds (16%) and organoheterocyclic compounds (10%) (Figure 1).

### 3.3 Prediction of PCOS‐Related Targets

A total of 910 metabolite targets were obtained from the SwissTargetPrediction database. A total of 297 disease targets were retrieved from DisGeNET and GeneCards databases. Fifty compounds were commonly identified by intersectional analysis using Venny 2.1 (Figure S4).

### 3.4 PPI Network Analysis

**FIGURE 2:**
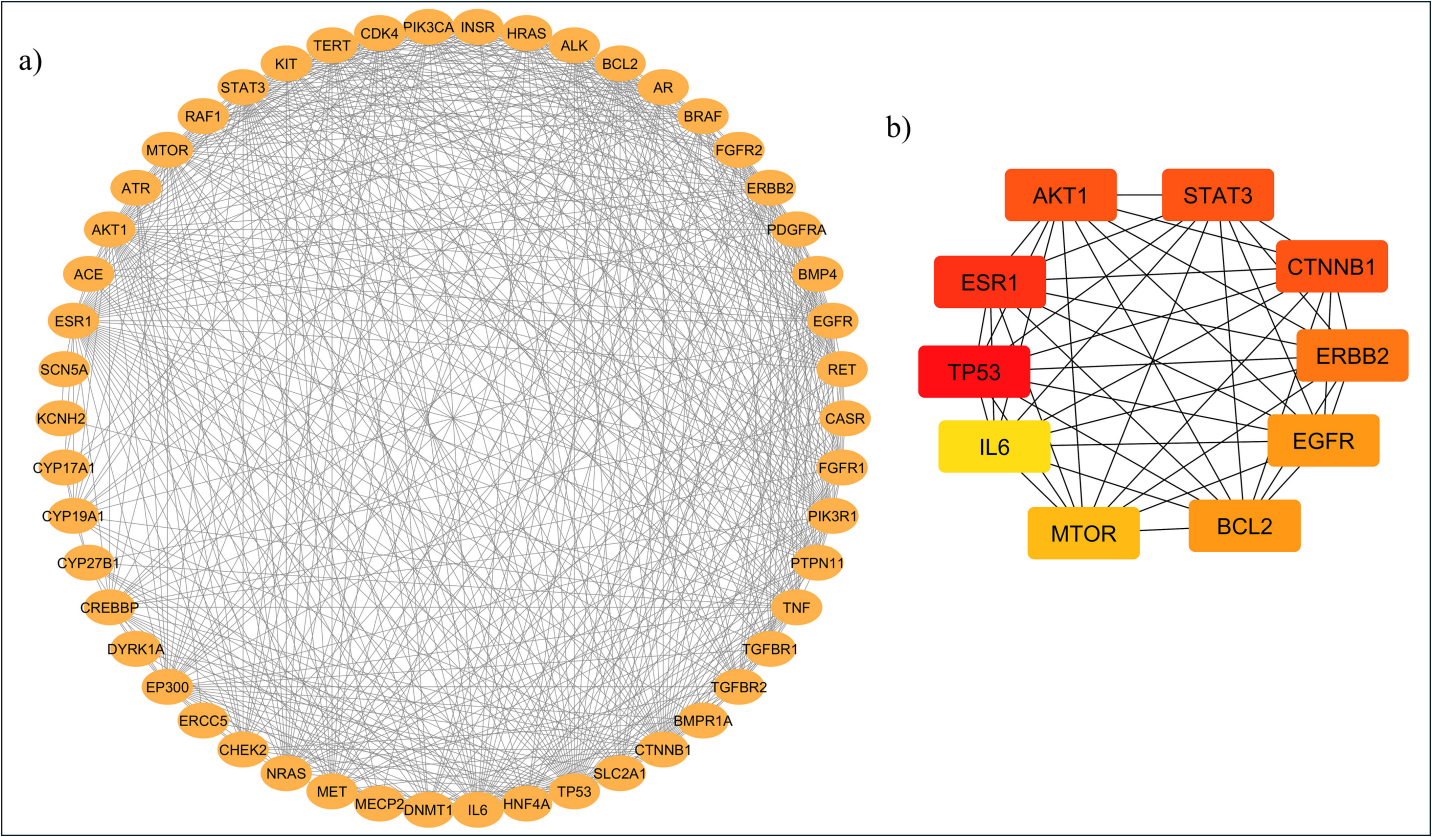
(a) Protein–Protein interaction (PPI) network of targets related to PCOS. (b) Hub targets with colour codes ranging from red to yellow (in terms of degree).

Fifty common genes were given as input to construct the PPI network. The PPI network consists of 50 nodes and 572 edges with a network density of 0.467 (Figure 2a). Cyto‐hubba plugin was used to determine the top ten hub targets (TP53, ESR1, AKT1, STAT3, CTNNB1, ERBB2, BCL2, EGFR, MTOR and IL6). The cyto‐hubba visualisation showed node colours ranging from red to yellow, with red representing greater degree values and yellow representing lower degree values (Figure 2b).

### 3.5 Functional Enrichment Analysis of Hub Targets

**FIGURE 3:**
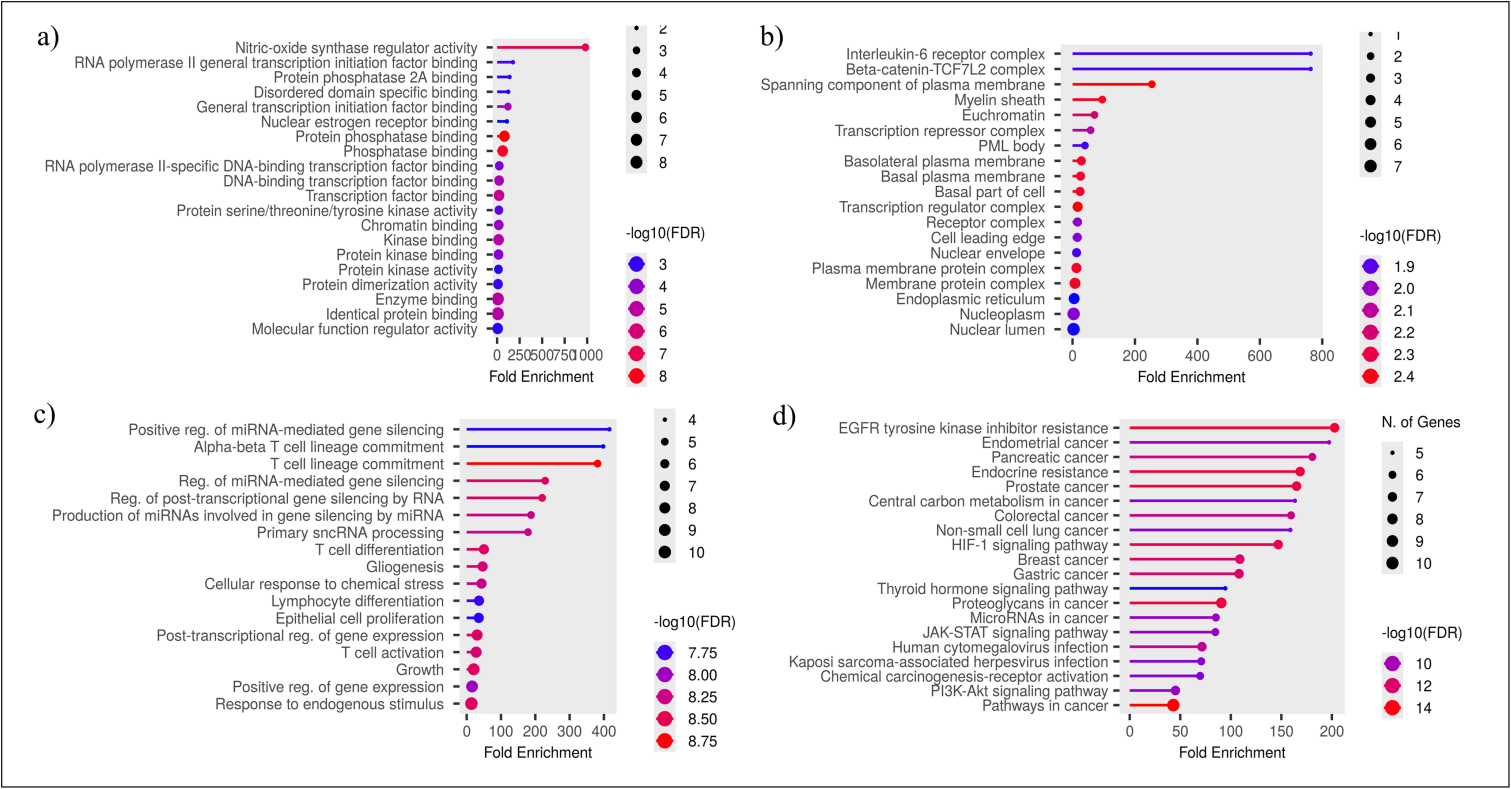
Bar plot representation of KEGG pathways and gene ontology (GO) for PCOS hub targets (a) GO analysis enriched in molecular process, (b) cellular process, (c) biological process and (d) KEGG pathway analysis of hub targets.

The top enriched pathways were ranked based on a false discovery rate cutoff of 0.05. GO and KEGG analysis results for the top hub targets are shown in Figure 3. The analysis revealed the involvement of biological processes such as positive regulation of post‐transcriptional gene silencing, T cell lineage commitment, lymphocyte differentiation and T cell differentiation. CC targets were involved in the interleukin‐6 receptor complex, the myelin sheath, transcription regulatory complexes, the transcription repressor complex and the plasma membrane protein complex. The primary molecular functions of the targets included nitric oxide synthase regulator activity, RNA polymerase II general transcription initiator factor binding, protein phosphatase 2A binding and nuclear oestrogen receptor binding. Figure 3a–c represents the top 20 enriched pathways for PCOS. KEGG analysis revealed pathways related to endometrial cancer, thyroid signalling pathway and endocrine resistance.

### 3.6 Drug Likeness Prediction and Molecular Docking Analysis

**FIGURE 4:**
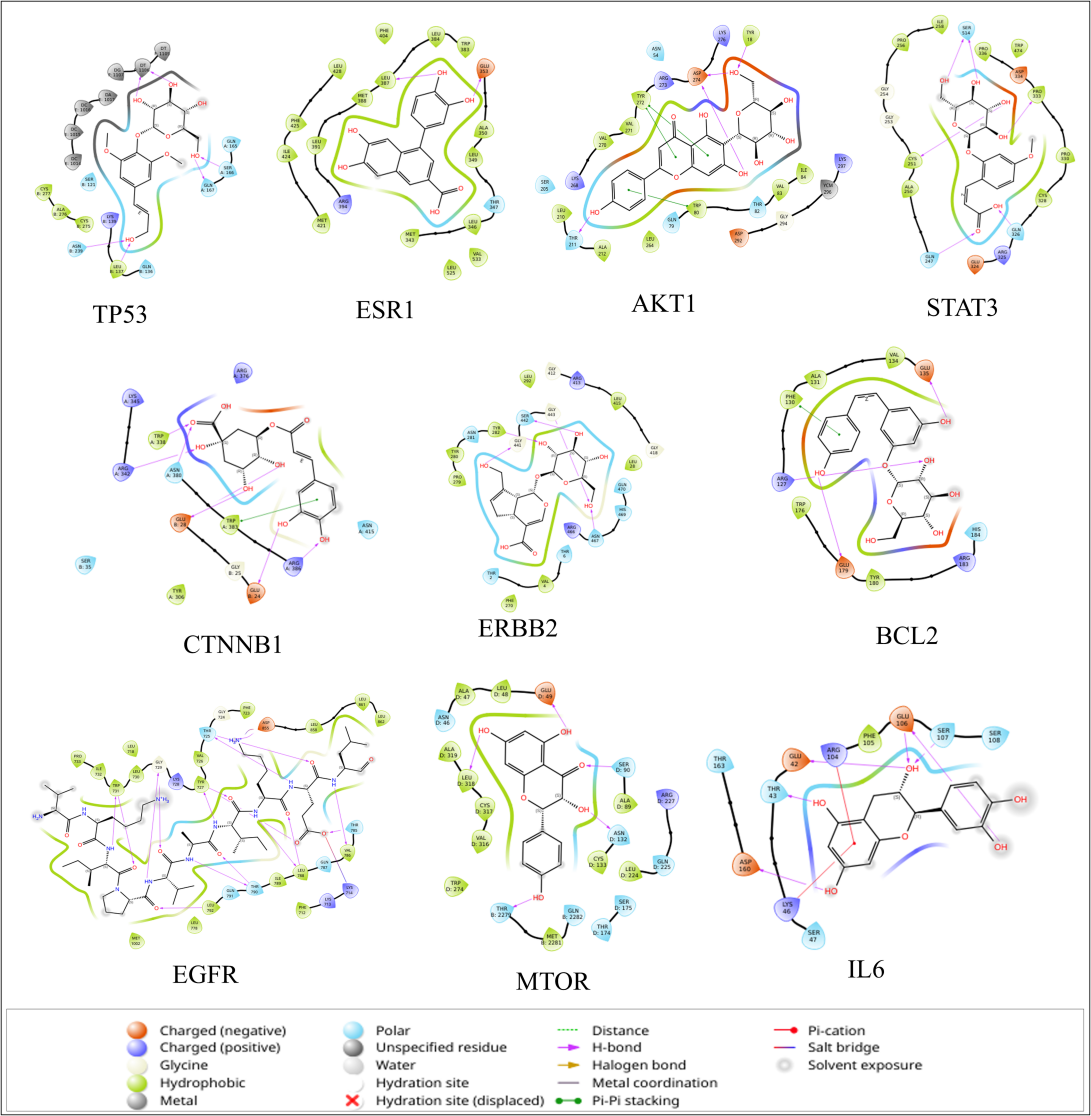
Two‐dimensional interaction diagram of the top hit ligand against PCOS hub targets.

Out of 186 metabolites identified using UHPLC–MS/MS, 122 metabolites were secondary metabolites. Drug likeness was predicted using the Qikprop tool, and the compounds were screened according to the Lipinski rule of five. After drug likeness prediction, 103 compounds (Table S3) were found to possess drug‐like properties. Molecular docking was performed for the 103 drug‐like compounds that were identified against the top hub targets. The hub targets' structures were chosen based on the lowest resolution. Each ligand was docked individually, and its binding affinity was assessed using the Glide score. Table 1 summarises the findings by presenting the Glide score and the best‐docked compound for each hub gene, and Figure 4 depicts the 2D interaction between the ligand and the protein targets. Notably, 4‐(3,4‐dihydroxyphenyl)‐6,7‐dihydroxynaphthalene‐2‐carboxylic acid had the strongest affinity for binding ESR1 (glide score: −12.968), followed by Isovitexin with AKT1 (−11.08) and Syringin with TP53 (−11.478). To validate the docking results, docking studies were conducted using the co‐crystallised ligand. The docking scores of the top‐scoring ligand and the co‐crystallised ligand with the targets were comparable. The results of co‐crystallised ligand docking are provided in Table S4. These docking results provide insight into the binding potential of the secondary metabolites to the key protein targets.

**TABLE 1:** Top ligand for each hub target and the interaction between the ligand and hub targets.

Hub targets	Compound name	Docking score	Interaction between the ligand and the amino acid of the target protein
TP53 (PDB ID: 1TUP)	Syringin	−11.478	H bond:‐ A:Ser166, A:Gln167, F:DT110, B:Asn239, B:leu137 Charged (positive):‐ B:Lys139 Polar:‐ B:Ser121, B:Asn239, B:Gln136, B:Gln165, A:Ser166, A:Gln167 Hydrophobic:‐ B:Cys277, B:Ala276, B:Cys275, B:leu137 Metal:‐ F:DT1105, F:DT1106, F:DG1107, E:DA1017, E:DC1016, E:DC1015, E:DC1014
ESR1 (PDB ID: 6VPF)	4‐(3,4‐dihydroxyphenyl)‐6,7‐dihydroxynaphthalene‐2‐carboxylic acid	−12.968	H bond:‐ Leu387, Glu353 Charged (negative):‐ Glu353 Charged (positive): Arg394 Polar:‐ Thr347 Hydrophobic:‐ Leu428, Phe425, Ile424, Met421, Leu391, Met388, Leu387, Leu384, Trp383, Phe404, Ala350, Leu349, Leu346, Met343, Val533, Leu525
AKT1 (PDB ID: 8UW7)	Isovitexin	−11.08	H bond:‐ Thr211, Tyr18, Asp274 Pi‐Pi stacking:‐ Trp80, Typ272 Charged (negative):‐Asp274, Asp 292 Charged (positive):‐ Lys268, Arg273, Lys276, Lys297 Polar: Ser205, Thr211, Asn54, Gln79, Thr82 Hydrophobic:‐ Leu210, Ala212, Val
STAT3 (PDB ID: 6NJS)	(Z)‐3‐[4‐methoxy‐2‐[(2S,3R,4S,5S,6R)‐3,4,5‐trihydroxy‐6‐(hydroxymethyl)oxan‐2‐yl]oxyphenyl]prop‐2‐enoic acid	−9.85	H bond:‐ Cys251, Gln247, Ser514, Pro333, Gln326 Charged (negative):‐ Asp334, Glu324 Charged (positive):‐ Arg325 Polar: Gln247, ser514, Gln326 Hydrophobic:‐ Ile258, Pro256, Cys251, Ala250, Pro336, Trp474, Pro333, Pro330, Cys328 Glycine:‐ Gly254, Gly253
CTNNB1 (PDB ID: 1JDH)	Chlorogenic acid	−6.242	H Bond:‐ A:Trp 338, A:Arg342, A:Asn380, A:Arg386, B:Glu24, B:Glu28 Pi—Pi stacking interactions:‐ A:Trp383 Charged (positive):‐ A:Arg386, A:Arg342, A:Lys345, A:Arg376 Charged (negative):‐ B:Glu28, B:Glu24 Polar:‐ A:Asn380, A:Asn415, B:Ser35 Hydrophobic:‐ A:Tyr306, A:Trp338, A:Trp383
ERBB2 (PDB ID: 2A91)	Geniposidic acid	−8.523	H bond:‐ Tyr282, Gly441, Ser442, Gly443, Asn467 Charged (positive): Arg413, Arg466 Polar:‐ Asn281, Ser442, Thr2, Thr6, Asn467, His469, Gln470 Hydrophobic:‐ Pro279, Tyr280, Tyr282, Leu292, Leu415, Leu28, Val4, Phe270 Glycine:‐ Gly441, Gly443, Gly412, Gly418
BCL2 (PDB ID: 8HTS)	(2S,3R,4S,5S,6R)‐2‐[3‐hydroxy‐5‐[(Z)‐2‐(4‐hydroxyphenyl)ethenyl]phenoxy]‐6‐(hydroxymethyl)oxane‐3,4,5‐triol	−6.124	H bond:‐ Arg127, Glu135, Glu179 Pi‐Pi stacking:‐ Phe130 Charged (negative):‐ Glu135, Glu179 Charged (positive):‐ Arg127, Arg182 Polar:‐ His184 Hydrophobic:‐ Phe130, Ala131, Val134, Trp176, Tyr180
EGFR (PDB ID: 3POZ)	Epigallocatechin	−7.073	H Bond:‐ Thr725, Tyr727, Gly729, Trp 731, Val786, Leu788, Thr790, Leu792, Lys714 Polar:‐ Thr725, Thr785, Gln787, Thr790, Gln791 Charged (positive):‐ Lys728, Lys713, Lys714 Charged (negative):‐ Asp855 Hydrophobic:‐ Pro733, Ile732, Trp731, Leu730, Leu718, Tyr727, Val726, Phe723, Leu858, Leu861, Leu862, Val786, Leu788, Ile789, Leu792, Leu778, Met1002, Phe712 Glycine:‐ Gly729, Gly724
MTOR (PDB ID: 4JSN)	(2S,3S)‐3,5,7‐trihydroxy‐2‐(4‐hydroxyphenyl)‐2,3‐dihydrochromen‐4‐one	−9.7	H bond:‐ D:Leu318, D:Glu49, D:Ser90, D:Asn132, B:Thr2279 Charged (negative):‐ D:Glu49 Charged (positive):‐ D:Arg227 Polar:‐ D:Asn46, D:Ser90, D:Asn132, B:Gln2282, B:Thr2279, D:Gln225, D:Ser175, D:Thr174 Hydrophobic:‐ D:Trp274, D:Val316, D:Cys317, D:Leu318, D:Ala319, D:Ala47, D:Leu48, D:Ala89, D:Cys133, B:Met2281, D:Leu224
IL6 (PDB ID: 1ALU)	Catechin	−6.556	H bond:‐ Asp160, Thr43, Glu42, Glu106, Ser107 Pi‐cation: Lys46, Arg104 Charged (negative):‐ Asp160, Glu42, Glu106 Charged (positive):‐ Lys46, Arg104 Polar:‐ Thr163, Ser47, Thr43, Ser107, Ser108 Hydrophobic:‐ Phe105

## 4 Discussion

*V. negundo* is a medicinal plant recognised for its benefits in managing PCOS. UPLC‐MS/MS analysis identified 186 bioactive compounds, which mainly consist of flavonoids, terpenoids and lignans. According to the literature, these compounds exhibit anti‐inflammatory and antioxidant properties (Mansuri et al. 2014). Although studies show that *V. negundo* has a therapeutic effect on PCOS, the mechanism of action remains unclear. To address this, our study employed network pharmacology to elucidate the mechanism and associated pathways.

Functional enrichment analysis revealed significant involvement of key pathways such as thyroid hormone signalling and endometrial cancer, both of which are closely associated with the pathophysiology of PCOS. PCOS is a recognised risk factor for endometrial cancer, primarily due to chronic anovulation and oestrogen‐driven endometrial hyperplasia (Li et al. 2022). The thyroid hormone signalling pathway is relevant because thyroid dysfunction has been associated with menstrual irregularities, ovulatory disorders and metabolic disturbances commonly observed in women with PCOS (Sheely and Pujare 2022). Similarly, the endocrine resistance pathway reflects altered hormone receptor signalling, which contributes to endocrine dysfunction in PCOS. The enrichment of the endometrial cancer pathway is also biologically meaningful, as women with PCOS have an increased risk of endometrial hyperplasia and endometrial cancer due to chronic anovulation and prolonged unopposed oestrogen exposure (Li et al. 2022). In addition, key hub genes such as IL‐6, ESR1 and CTNNB play a crucial role in regulating multiple factors of PCOS, including chronic inflammation, metabolic disturbances and hormonal imbalances. This demonstrates the multitarget potential of genes, rather than limiting them to a single‐target model.

To identify potential therapeutic candidates, molecular docking studies were conducted with active metabolites derived from *V. negundo* . Among the lead compounds, 4‐(3,4‐dihydroxyphenyl)‐6,7‐dihydroxynaphthalene‐2‐carboxylic acid exhibited a high binding affinity to ESR1, the oestrogen receptor alpha gene. ESR1 plays a critical role in follicular development and ovulation, and its modulation could be a promising strategy to restore normal ovarian function in PCOS (Muccee et al. 2024). Additionally, Isovitexin, a flavonoid compound identified in the plant, demonstrated strong binding affinity with AKT1, a gene whose overexpression has been reported in hyperandrogenic PCOS cases (Nekoonam et al. 2017). This suggests a possible regulatory effect of Isovitexin on AKT1 expression, thereby contributing to the correction of downstream signalling pathways dysregulated in PCOS. Taken together, these findings suggest that bioactive compounds in *V. negundo* may exert therapeutic effects in PCOS through multitarget modulation involving hormonal, metabolic and proliferative signalling pathways. However, the pharmacological relevance of these interactions must be further validated through in vitro and in vivo studies to confirm their efficacy and safety.

## 5 Conclusion

The present study elucidates the mechanism of action of bioactive compounds from *V. negundo* in the management of PCOS. Metabolite profiling revealed 186 metabolites, including 122 secondary metabolites. PPI analysis identified the top 10 key PCOS‐associated targets. Functional enrichment analysis pointed to critical pathways such as endometrial cancer and thyroid hormone signalling associated with PCOS. In silico molecular docking highlighted promising compounds—Syringin, 4‐(3,4‐dihydroxyphenyl)‐6,7‐dihydroxynaphthalene‐2‐carboxylic acid, Isovitexin, and (2S,3S)‐3,5,7‐trihydroxy‐2‐(4‐hydroxyphenyl)‐2,3‐dihydrochromen‐4‐one. These findings suggest potential therapeutic candidates for PCOS, meriting further validation through in vitro and in vivo studies.

## Author Contributions

**Nishmitha R. Poojary:** writing – original draft, investigation, formal analysis, software data curation. **Neeharika Narisepalli Venkatasai:** writing – original draft, investigation, visualisation, formal analysis, software. **Kannath U. Sanjay:** writing – original draft, investigation, formal analysis, software. **Supraja M. Kodanch:** investigation, data curation, visualisation. **Padmalatha S. Rai:** conceptualisation, methodology, supervision, resources, writing – review and editing, project administration.

## Funding

The authors thank the Manipal Academy of Higher Education, Manipal, India, for financial support.

## Conflicts of Interest

The authors declare no conflicts of interest.

## Supporting information


**Figure S1:** Herbarium voucher specimen of *Vitex negundo* L.
**Figure S2:** Agarose gel electrophoretic image of PCR amplicon with *nrITS* marker of *Vitex negundo* L. Lane 1. 100 bp DNA ladder Lane 2. PCR amplicon Lane 3. Negative control.
**Figure S3:** Clustal W alignment of *Vitex negundo* L. using nrITS marker.
**Figure S4:** Venn diagram showing the overlapping targets between compound targets and PCOS targets.
**Table S1:** PCR conditions for DNA barcoding using *nrITS* markers.
**Table S2:** List of putative metabolites obtained after UPLC‐MS/MS analysis.
**Table S3:** List of metabolites used for molecular docking studies.
**Table S4:** Molecular docking of co‐crystallised ligand with hub targets.

## Data Availability

Data will be made available upon request.
