# Supplementary material for: Elucidating the Molecular Mechanisms of Vitex negundo L. in Polycystic Ovary Syndrome via Metabolomics‐Driven Network Pharmacology
Source: Biomed Chromatogr. 2026 Aug 10;40(9):e70592. doi: 10.1002/bmc.70592 (PMC13456947; doi:10.1002/bmc.70592)
Supplement: Supplementary file 1 [file BMC-40-e70592-s001.docx]

**Supplementary Material**

**Elucidating the Molecular Mechanisms of *Vitex negundo* L. in Polycystic Ovary Syndrome via Metabolomics-Driven Network Pharmacology**

Nishmitha R Poojary^a^, Neeharika Narisepalli Venkatasai^a^, [Kannath U Sanjay](https://link.springer.com/article/10.1007/s00425-025-04637-w#auth-Kannath_U_-Sanjay-Aff1)^a^, Supraja M Kodanch^a^, Padmalatha S Rai^a*^

^a^ Department of Biotechnology, Manipal School of Life Sciences, Manipal Academy of Higher Education, Manipal, Karnataka, India

^*^Corresponding author

Department of Biotechnology, Manipal School of Life Sciences

Manipal Academy of Higher Education, Manipal – 576104, Karnataka, India

Tele: +91 820 2923505, +91 820 2922058

Fax Numbers: +91 820 2571919

Email: padmalatha.rai@manipal.edu

ORCiD id: 0000-0001-7159-0560

# **List of figures**

**Figure S1** Herbarium voucher specimen of *Vitex negundo* L.

**Figure S2** Agarose gel electrophoretic image of PCR amplicon with *nrITS* marker of *Vitex negundo* L.

Lane 1. 100 bp DNA ladder Lane 2. PCR amplicon Lane 3. Negative control

**Figure S3** Clustal W alignment of Vitex negundo L. using nrITS marker

**Figure S4** Venn diagram showing the overlapping targets between compound targets and PCOS targets

# **List of tables**

**Table S1** PCR conditions for DNA barcoding using *nrITS* markers

| Steps | Temperature | Time |  |
| --- | --- | --- | --- |
| Initial Denaturation | 95°C | 5 minutes | 30 cycles |
| Denaturation | 95°C | 1 minute |  |
| Annealing | 55°C | 45 seconds |  |
| Extension | 72°C | 1 minute 30 seconds |  |
| Final Extension | 72°C | 10 minutes |  |
| Holding | 4°C | ∞ |  |

**Table S2** List of putative metabolites obtained after UPLC–MS/MS analysis

| Metabolite name | Retention time | Precursor m/z | Theoretical m/z | PPM Error | Adduct | Formula | MS/MS fragments |
| --- | --- | --- | --- | --- | --- | --- | --- |
| (-)-Epicatechin | 26.048 | 289.06894 | 289.0717622 | 9.76 | [M-H]- | C15H14O6 | 289.0689, 290.0722, 291.0756 |
| (-)-Riboflavin | 17.424 | 375.12891 | 375.1310084 | 5.59 | [M-H]- | C17H20N4O6 | 375.1289, 376.1322, 377.1356 |
| (1R,2R,4aS,6aS,6bR,9R,10R,11R,12aR)-1,10,11-trihydroxy-9-(hydroxymethyl)-1,2,6a,6b,9,12a-hexamethyl-2,3,4,5,6,6a,7,8,8a,10,11,12,13,14b-tetradecahydropicene-4a-carboxylic acid | 26.915 | 503.33813 | 503.3378133 | 0.62 | [M-H]- | C30H48O6 | 503.3381, 504.3414, 505.3448 |
| (1R,3R,4S,5R)-1,3,4-trihydroxy-5-[(E)-3-(4-hydroxy-3-methoxyphenyl)prop-2-enoyl]oxycyclohexane-1-carboxylic acid | 22.795 | 367.10269 | 367.1034562 | 2.08 | [M-H]- | C17H20O9 | 367.1026, 368.1059, 369.1093 |
| (1S,4aS,7R,7aS)-7-hydroxy-7-[[(E)-3-(4-hydroxyphenyl)prop-2-enoyl]oxymethyl]-1-[(2S,3R,4S,5S,6R)-3,4,5-trihydroxy-6-(hydroxymethyl)oxan-2-yl]oxy-4a,7a-dihydro-1H-cyclopenta[c]pyran-4-carboxylic acid | 22.447 | 535.14551 | 535.1457149 | 0.38 | [M-H]- | C25H28O13 | 535.1455, 536.1488, 537.1522 |
| (1S,4aS,7S,7aS)-7-hydroxy-7-methyl-1-[(2S,3R,4S,5S,6R)-3,4,5-trihydroxy-6-(hydroxymethyl)oxan-2-yl]oxy-4a,5,6,7a-tetrahydro-1H-cyclopenta[c]pyran-4-carboxylic acid | 19.37 | 375.129 | 375.129671 | 1.78 | [M-H]- | C16H24O10 | 375.1290,376.1323, 377.1357 |
| (1S,4aS,7S,7aS)-7-hydroxy-7-methyl-1-[(2S,3R,4S,5S,6R)-3,4,5-trihydroxy-6-[(4-hydroxybenzoyl)oxymethyl]oxan-2-yl]oxy-4a,5,6,7a-tetrahydro-1H-cyclopenta[c]pyran-4-carboxylic acid | 21.315 | 495.14999 | 495.1508003 | 1.63 | [M-H]- | C23H28O12 | 495.1499, 496.1533, 497.1567 |
| (2R,3S,4S,5R,6S)-2-(hydroxymethyl)-6-[4-prop-2-enyl-2-[(2S,3R,4S,5S,6R)-3,4,5-trihydroxy-6-(hydroxymethyl)oxan-2-yl]oxyphenoxy]oxane-3,4,5-triol | 21.452 | 497.1618 | 497.1629444 | 2.30 | [M+Na]+ | C21H30O12 | 497.1618, 498.1651, 499.1685 |
| (2S,3R,4S,5S,6R)-2-[3-hydroxy-5-[(Z)-2-(4-hydroxyphenyl)ethenyl]phenoxy]-6-(hydroxymethyl)oxane-3,4,5-triol | 22.619 | 389.12363 | 389.1241917 | 1.44 | [M-H]- | C20H22O8 | 389.1236, 390.1269, 391.1303 |
| (2S,3S)-2-(3,4,5-trihydroxyphenyl)-3,4-dihydro-2H-chromene-3,5,7-triol | 4.521 | 305.06369 | 305.0666768 | 9.79 | [M-H]- | C15H14O7 | 305.0636,306.0670, 307.0704 |
| (2S,3S)-3,5,7-trihydroxy-2-(4-hydroxyphenyl)-2,3-dihydrochromen-4-one | 23.361 | 289.07001 | 289.0706641 | 2.26 | [M+H]+ | C15H12O6 | 289.0700, 290.0733, 291.0767 |
| (2S,3S,4S,5R,6S)-3,4,5-trihydroxy-6-(5-hydroxy-4-oxo-2-phenylchromen-7-yl)oxyoxane-2-carboxylic acid | 21.709 | 431.0961 | 431.0972728 | 2.72 | [M+H]+ | C21H18O10 | 431.096, 432.0994,433.1028 |
| (5E)-4-[(E)-3-(4-hydroxyphenyl)prop-2-enoyl]oxy-2-methyl-5-[2-[(2R,3R,4S,5S,6R)-3,4,5-trihydroxy-6-(hydroxymethyl)oxan-2-yl]oxyethylidene]cyclopentene-1-carboxylic acid | 22.612 | 491.15561 | 491.1558857 | 0.56 | [M-H]- | C24H28O11 | 491.1556, 492.1589, 493.1623 |
| (E)-3-[4-[(2S,3R,4S,5S,6R)-3,4,5-trihydroxy-6-(hydroxymethyl)oxan-2-yl]oxyphenyl]prop-2-enoic acid | 20.125 | 349.08875 | 349.0893855 | 1.82 | [M+Na]+ | C15H18O8 | 349.0879, 350.0913, 351.0946 |
| (E)-N-[2-hydroxy-2-(4-hydroxyphenyl)ethyl]-3-(4-hydroxy-3-methoxyphenyl)prop-2-enamide | 18.959 | 330.13242 | 330.1335987 | 3.57 | [M+H]+ | C18H19NO5 | 330.1324, 331.1357,332.1391 |
| (Z)-3-[4-methoxy-2-[(2S,3R,4S,5S,6R)-3,4,5-trihydroxy-6-(hydroxymethyl)oxan-2-yl]oxyphenyl]prop-2-enoic acid | 22.056 | 374.14288 | 374.1445552 | 4.47 | [M+NH4]+ | C16H20O9 | 374.1428, 375.1462, 376.1495 |
| [(1aS,1bS,2S,5aR,6S,6aS)-1a-(hydroxymethyl)-2-[(2S,3R,4S,5S,6R)-3,4,5-trihydroxy-6-(hydroxymethyl)oxan-2-yl]oxy-2,5a,6,6a-tetrahydro-1bH-oxireno[5,6]cyclopenta[1,3-c]pyran-6-yl] (E)-3-(3-hydroxy-4-methoxyphenyl)prop-2-enoate | 23.056 | 537.16064 | 537.161365 | 1.34 | [M-H]- | C25H30O13 | 537.1606, 538.1639, 539.1673 |
| [(1R,2R,3aR,5R,6E,10R,11S,13R,13aS)-1,2,3a,11,13-pentaacetyloxy-2,5,8,8-tetramethyl-12-methylidene-4,9-dioxo-3,5,10,11,13,13a-hexahydro-1H-cyclopenta[12]annulen-10-yl] 2-methylbutanoate | 25.078 | 691.29596 | 691.2971302 | 1.69 | [M-H]- | C35H48O14 | 691.2959, 692.2993, 693.3026 |
| [(2S,3R,4R,5R,6S)-2-[2-(3,4-dihydroxyphenyl)-5,7-dihydroxy-4-oxochromen-3-yl]oxy-4,5-dihydroxy-6-methyloxan-3-yl] 3,4,5-trihydroxybenzoate | 20.085 | 599.10321 | 599.1042441 | 1.72 | [M-H]- | C28H24O15 | 599.1032, 600.1065, 601.1099 |
| [3-hydroxy-4-[(2S,3R,4S,5S,6R)-3,4,5-trihydroxy-6-(hydroxymethyl)oxan-2-yl]oxyphenyl]methyl 3,4-dihydroxybenzoate | 22.665 | 439.12012 | 439.1234875 | 7.66 | [M+H]+ | C20H22O11 | 439.1201, 440.1234,441.1268 |
| [6]-Gingerol | 27.277 | 293.17856 | 293.1758333 | 9.30 | [M-H]- | C17H26O4 | 293.1785, 294.1819, 295.1852 |
| 11-hydroxy-9-(hydroxymethyl)-2-methoxycarbonyl-2,6a,6b,9,12a-pentamethyl-10-(3,4,5-trihydroxyoxan-2-yl)oxy-1,3,4,5,6,6a,7,8,8a,10,11,12,13,14b-tetradecahydropicene-4a-carboxylic acid | 23.801 | 687.37024 | 687.3714806 | 1.80 | [M+Na]+ | C36H56O11 | 687.3702, 688.3735, 689.3769 |
| 13-HODE | 29.156 | 295.22693 | 295.2278689 | 3.18 | [M-H]- | C18H32O3 | 295.2269, 296.2302,297.2336 |
| 2-(3,4-dimethoxyphenyl)-5,7-dihydroxy-3,6-dimethoxy-4H-chromen-4-one | 26.743 | 373.09207 | 373.0928915 | 2.20 | [M-H]- | C19H18O8 | 373.0920,374.0954,375.0987 |
| 2-(3-hydroxy-5-methoxyphenoxy)-6-(hydroxymethyl)oxane-3,4,5-triol | 16.879 | 301.09241 | 301.0928915 | 1.59 | [M-H]- | C13H18O8 | 301.0924,302.0957, 303.0991 |
| 2',5'-Dihydroxy-4-Methoxychalcone | 24.069 | 271.09583 | 271.0964849 | 2.41 | [M+H]+ | C16H14O4 | 271.0958,272.0991, 273.1025 |
| 2-[(2S,4aR,8aS)-2-hydroxy-4a-methyl-8-methylidene-3,4,5,6,7,8a-hexahydro-1H-naphthalen-2-yl]prop-2-enoic acid | 30.707 | 249.14891 | 249.1496186 | 2.84 | [M-H]- | C15H22O3 | 249.1489,250.1522,251.1556 |
| 2-[4-[3-[3,4-dihydroxy-4-(hydroxymethyl)oxolan-2-yl]oxy-4,5-dihydroxy-6-(hydroxymethyl)oxan-2-yl]oxyphenyl]-7-hydroxy-2,3-dihydrochromen-4-one | 23.318 | 549.16125 | 549.161365 | 0.20 | [M-H]- | C26H30O13 | 549.1612,550.1646,551.1679 |
| 2-methyl-3-[(2S,3R,4S,5S,6R)-3,4,5-trihydroxy-6-(hydroxymethyl)oxan-2-yl]oxypyran-4-one | 17.465 | 311.07263 | 311.0737355 | 3.55 | [M+Na]+ | C12H16O8 | 311.0731,312.0764,313.0798 |
| 3,4-di-O-caffeoylquinic acid | 22.143 | 517.1311 | 517.1340522 | 5.70 | [M+H]+ | C25H24O12 | 517.1311,518.1344,519.1378 |
| 3-[4,5-dihydroxy-6-(hydroxymethyl)-3-[3,4,5-trihydroxy-6-(hydroxymethyl)oxan-2-yl]oxyoxan-2-yl]oxy-5,7-dihydroxy-2-(4-hydroxyphenyl)chromen-4-one | 21.315 | 609.14172 | 609.1461089 | 7.20 | [M-H]- | C27H30O16 | 609.1417,610.1450,611.1484 |
| 3-p-Coumaroylquinic acid | 21.315 | 337.09183 | 337.0928915 | 3.14 | [M-H]- | C16H18O8 | 337.0918,338.0951, 339.0985 |
| 4-(3,4-dihydroxyphenyl)-6,7-dihydroxynaphthalene-2-carboxylic acid | 22.524 | 311.05511 | 311.0561121 | 3.22 | [M-H]- | C17H12O6 | 311.0551, 312.0584,313.0618 |
| 4-[(E)-3-[1-carboxy-2-(3,4-dihydroxyphenyl)ethoxy]-3-oxoprop-1-enyl]-2-(3,4-dihydroxyphenyl)-7-hydroxy-2,3-dihydro-1-benzofuran-3-carboxylic acid | 22.185 | 537.10016 | 537.1038501 | 6.87 | [M-H]- | C27H22O12 | 537.1001,538.1035, 539.1068 |
| 4-Methylabyssinone V | 24.99 | 421.20679 | 421.2020481 | 11.25 | [M-H]- | C26H30O5 | 421.2067,422.2101,423.2135 |
| 5,10-dimethoxy-2,2-dimethylpyrano[3,2-g]chromen-8-one | 16.25 | 287.09015 | 287.0924976 | 8.17 | [M-H]- | C16H16O5 | 287.0901, 288.0935, 289.0968 |
| 5,7-dihydroxy-2-(4-hydroxyphenyl)-3,6-dimethoxy-4H-chromen-4-one | 27.262 | 329.06601 | 329.0666768 | 2.02 | [M-H]- | C17H14O7 | 329.0660,330.0694,331.0727 |
| 5,7-dihydroxy-2-(4-hydroxyphenyl)-6-[3,4,5-trihydroxy-6-(hydroxymethyl)oxan-2-yl]chromen-4-one | 22.491 | 433.11139 | 433.1129228 | 3.53 | [M+H]+ | C21H20O10 | 433.1113,434.1147,435.1181 |
| 5,7-dihydroxy-3-[(2S,3R,4S,5R,6R)-3,4,5-trihydroxy-6-(hydroxymethyl)oxan-2-yl]oxy-2-(3,4,5-trihydroxyphenyl)chromen-4-one | 24.552 | 479.08218 | 479.0831147 | 1.95 | [M-H]- | C21H20O13 | 479.0821,480.0855,481.0888 |
| 5,7-Dihydroxyflavanone | 20.523 | 255.0657 | 255.0662829 | 2.28 | [M-H]- | C15H12O4 | 255.0657,256.0690,257.0724 |
| 5-[(2R,3S)-6-hydroxy-2-(4-hydroxyphenyl)-4-[(E)-2-(4-hydroxyphenyl)ethenyl]-2,3-dihydro-1-benzofuran-3-yl]benzene-1,3-diol | 21.494 | 453.14047 | 453.1343624 | 13.47 | [M-H]- | C28H22O6 | 453.1404,454.1438,455.1471 |
| 5-aminoimidazole-4-carboxamide-1-beta-d-ribofuranosyl 5'-monophosphate | 4.693 | 337.05307 | 337.0554745 | 7.13 | [M-H]- | C9H15N4O8P | 337.0530,338.0564,339.0597 |
| 5-hydroxy-3,6,7-trimethoxy-2-(4-methoxyphenyl)-4H-chromen-4-one | 4.679 | 357.10254 | 357.0979769 | 12.77 | [M-H]- | C19H18O7 | 357.1025,358.1058, 359.1092 |
| 7-[(E)-3-methyl-4-(4-methyl-5-oxo-2H-furan-2-yl)but-2-enoxy]chromen-2-one | 5.342 | 349.10968 | 349.1046417 | 14.43 | [M+Na]+ | C19H18O5 | 349.1096,350.1130,351.1163 |
| 9-hydroxy-1,4a-dimethyl-7-propan-2-yl-2,3,4,9,10,10a-hexahydrophenanthrene-1-carboxylic acid | 24.772 | 317.20972 | 317.2111208 | 4.41 | [M+H]+ | C20H28O3 | 317.2097,318.2130,319.2164 |
| 9-hydroxy-10,12-octadecadienoic acid | 29.156 | 295.22693 | 295.2278689 | 3.18 | [M-H]- | C18H32O3 | 295.2269,296.2302,297.2336 |
| 9-Kode | 28.718 | 293.21185 | 293.2122188 | 1.25 | [M-H]- | C18H30O3 | 293.2118, 294.2152,295.2185 |
| 9-Methoxy-2,2-dimethyl-2,6-dihydro-pyrano[3,2-c]quinolin-5-one | 4.636 | 258.10925 | 258.1124693 | 12.47 | [M+H]+ | C15H15NO3 | 258.1092,259.1126,260.1159 |
| Abietic acid | 28.762 | 303.23093 | 303.2318562 | 3.05 | [M+H]+ | C20H30O2 | 303.2309,304.2342,305.2376 |
| Agnuside | 21.793 | 484.18021 | 484.1813346 | 2.32 | [M+NH4]+ | C22H26O11 | 484.1789,485.1823,486.1857 |
| Albiflorin | 22.437 | 479.15518 | 479.1558857 | 1.47 | [M-H]- | C23H28O11 | 479.1551,480.1585,481.1618 |
| Aloenin | 20.157 | 409.11118 | 409.1140209 | 6.94 | [M-H]- | C19H22O10 | 409.1111,410.1145,411.1178 |
| Arabinofuranosyluracil | 13.547 | 243.06146 | 243.0622601 | 3.29 | [M-H]- | C9H12N2O6 | 243.0614,244.0647,245.0681 |
| Arbutin | 19.544 | 271.08182 | 271.0823269 | 1.86 | [M-H]- | C12H16O7 | 271.0818,272.0851,273.0885 |
| Arctiin | 23.624 | 535.21362 | 535.2173879 | 7.03 | [M+H]+ | C27H34O11 | 535.2136,536.2169,537.2203 |
| Asiatic Acid | 28.377 | 487.34216 | 487.3428986 | 1.51 | [M-H]- | C30H48O5 | 487.3421,488.3455,489.3488 |
| Aucubin | 16.968 | 345.11798 | 345.1191063 | 3.26 | [M-H]- | C15H22O9 | 345.1179,346.1213,347.1246 |
| Bakuchiol | 30.494 | 257.18906 | 257.1899914 | 3.62 | [M+H]+ | C18H24O | 257.1890,258.1924,259.1957 |
| Bicuculline (+) | 21.622 | 368.11157 | 368.1128633 | 3.51 | [M+H]+ | C20H17NO6 | 368.1115,369.1149, 370.1182 |
| Bungeiside C | 4.651 | 429.14066 | 429.1402356 | 0.98 | [M-H]- | C19H26O11 | 429.1406,430.1440,431.1473 |
| Cafestol | 24.772 | 317.20972 | 317.2111208 | 4.41 | [M+H]+ | C20H28O3 | 317.2097,318.2130,319.2164 |
| Candidone | 24.56 | 351.1651 | 351.1601832 | 14.0 | [M-H]- | C22H24O4 | 351.1651,352.1684,353.1718 |
| Casticin | 26.787 | 375.10571 | 375.1074435 | 4.62 | [M+H]+ | C19H18O8 | 375.1057,376.1090,377.1124 |
| Catechin | 26.048 | 289.06894 | 289.0717622 | 9.76 | [M-H]- | C15H14O6 | 289.0689,290.0722 291.0756 |
| Cearoin | 24.203 | 243.06877 | 243.0662829 | 10.23 | [M-H]- | C14H12O4 | 243.0687, 244.0721,245.0754 |
| Chloramphenicol | 23.056 | 321.00409 | 321.0050509 | 2.99 | [M-H]- | C11H12Cl2N2O5 | 321.0041, 322.0075,323.0108 |
| Chlorogenic acid | 22.185 | 353.0871 | 353.0878062 | 1.99 | [M-H]- | C16H18O9 | 353.0871,354.0904,355.0938 |
| Claussequinone | 21.792 | 287.09018 | 287.0913995 | 4.24 | [M+H]+ | C16H14O5 | 287.0901,288.0935,289.0968 |
| Coniferin | 20.122 | 360.16464 | 360.1652907 | 1.80 | [M+NH4]+ | C16H22O8 | 360.1646,361.1679,362.1713 |
| Cytidine | 4.65 | 242.07892 | 242.0782445 | 2.79 | [M-H]- | C9H13N3O5 | 242.0789,243.0822,244.0856 |
| D-(+)-Pantothenic acid | 18.216 | 218.10233 | 218.1033966 | 4.89 | [M-H]- | C9H17NO5 | 218.1023,219.1057,220.1090 |
| Deacetoxy-7-Oxogedunin | 20.92 | 456.24146 | 456.2380617 | 7.44 | [M+NH4]+ | C26H30O6 | 456.2414, 457.2448,458.2481 |
| Dehydroabietic acid | 30.494 | 301.21484 | 301.2162061 | 4.53 | [M+H]+ | C20H28O2 | 301.2148,302.2181,303.2215 |
| Dehydrorotenone | 15.678 | 393.13556 | 393.1332644 | 5.83 | [M+H]+ | C23H20O6 | 393.1355,394.1389,395.1422 |
| D-Glucose 1-phosphate | 24.983 | 259.02402 | 259.022443 | 6.08 | [M-H]- | C6H13O9P | 259.0240,260.0273,261.0307 |
| D-Glucose-6-phosphate | 24.906 | 259.02441 | 259.022443 | 7.59 | [M-H]- | C6H13O9P | 259.0244,260.0277,261.0311 |
| Dihydrosphingosine | 26.447 | 302.30453 | 302.3053555 | 2.73 | [M+H]+ | C18H39NO2 | 302.3045,303.3078,304.3112 |
| Duguetine | 18.97 | 356.15408 | 356.1492488 | 13.56 | [M+H]+ | C20H21NO5 | 356.1540,357.1574,358.1607 |
| Ellagic acid | 15.766 | 303.0152 | 303.0135432 | 5.46 | [M+H]+ | C14H6O8 | 303.0152, 304.0185,305.0219 |
| Epigallocatechin | 4.521 | 305.06369 | 305.0666768 | 9.79 | [M-H]- | C15H14O7 | 305.0636,306.0670,307.0704 |
| Epigallocatechin gallate | 15.821 | 457.07913 | 457.0776354 | 3.26 | [M-H]- | C22H18O11 | 457.0791,458.0824,459.0858 |
| Eriodictyol 7,3'-dimethyl ether | 26.873 | 317.10104 | 317.1019642 | 2.91 | [M+H]+ | C17H16O6 | 317.1010,318.1043,319.1077 |
| Eriodictyol | 24.023 | 287.05554 | 287.0561121 | 1.99 | [M-H]- | C15H12O6 | 287.0555,288.0588,289.0622 |
| Esculin | 19.37 | 339.07135 | 339.0721561 | 2.37 | [M-H]- | C15H16O9 | 339.0712,340.0746,341.0779 |
| Eudesmin | 26.614 | 387.17844 | 387.1802145 | 4.58 | [M+H]+ | C22H26O6 | 387.1784,388.1817,389.1851 |
| Feruloyltyramine | 19.676 | 314.13763 | 314.1386841 | 3.35 | [M+H]+ | C18H19NO4 | 314.1376, 315.1409,316.1443 |
| Fisetin | 25.4 | 287.0542 | 287.055014 | 2.83 | [M+H]+ | C15H10O6 | 287.0542,288.0575, 289.0609 |
| Formononetin | 26.967 | 269.07755 | 269.0808349 | 12.20 | [M+H]+ | C16H12O4 | 269.0775,270.0809,271.0842 |
| Gamma-mangostin | 16.517 | 395.15479 | 395.1500125 | 12.09 | [M-H]- | C23H24O6 | 395.1547,396.1581,397.1615 |
| Geniposidic acid | 17.602 | 373.11337 | 373.1140209 | 1.74 | [M-H]- | C16H22O10 | 373.1133,374.1167,375.1200 |
| Gentiobiose | 5.078 | 360.14932 | 360.1500345 | 1.98 | [M+NH4]+ | C12H22O11 | 360.1493,361.1526,362.1560 |
| Ginkgolide B | 23.003 | 425.14044 | 425.144223 | 8.89 | [M+H]+ | C20H24O10 | 425.1404,426.1437,427.1471 |
| Goniothalenol | 4.181 | 233.07782 | 233.0808349 | 12.93 | [M+H]+ | C13H12O4 | 233.0778,234.0811,235.0845 |
| Gossypin | 24.552 | 479.08218 | 479.0831147 | 1.95 | [M-H]- | C21H20O13 | 479.0821,480.0855, 481.0888 |
| Goyazensolide | 26.369 | 361.12689 | 361.128179 | 3.56 | [M+H]+ | C19H20O7 | 361.1268, 363.1336 |
| Grayanotoxin I | 27.606 | 411.2377 | 411.2388275 | 2.74 | [M-H]- | C22H36O7 | 411.2377, 412.2410, 413.2444 |
| Hederagenin | 30.438 | 471.34717 | 471.347984 | 1.72 | [M-H]- | C30H48O4 | 471.3471, 472.3505, 473.3538 |
| Heralenol | 22.143 | 305.10101 | 305.1019642 | 3.12 | [M+H]+ | C16H16O6 | 305.1010, 306.1043, 307.1077 |
| Homoorientin | 21.751 | 447.09186 | 447.0932855 | 3.18 | [M-H]- | C21H20O11 | 447.0920, 448.0954, 449.0987 |
| Hyperoside | 23.321 | 463.08731 | 463.0882001 | 1.92 | [M-H]- | C21H20O12 | 463.0873, 464.0906, 465.0940 |
| Irigenin | 25.961 | 359.07657 | 359.0772415 | 1.86 | [M-H]- | C18H16O8 | 359.0766, 360.0799, 361.0833 |
| Irisxanthone | 22.967 | 435.09174 | 435.0932855 | 3.55 | [M-H]- | C20H20O11 | 435.0917, 436.0950, 437.0984 |
| Isoquercitrin | 23.321 | 463.08731 | 463.0882001 | 1.92 | [M-H]- | C21H20O12 | 463.0873, 464.0906, 465.0940 |
| Isorhapontin | 17.334 | 419.13263 | 419.1347563 | 5.07 | [M-H]- | C21H24O9 | 419.1326, 420.1359, 421.1393 |
| Isosakuranetin | 21.792 | 287.09018 | 287.0913995 | 4.24 | [M+H]+ | C16H14O5 | 287.0901, 288.0935, 289.0968 |
| Isotaxiresinol | 25.256 | 345.13376 | 345.1343624 | 1.74 | [M-H]- | C19H22O6 | 345.1337, 346.1371, 347.1404 |
| Isovitexin | 22.491 | 455.09268 | 455.0948648 | 4.80 | [M+Na]+ | C21H20O10 | 455.0926, 456.0960, 457.0993 |
| Jaceidin | 25.918 | 361.09091 | 361.0917935 | 2.44 | [M+H]+ | C18H16O8 | 361.0909, 362.0942, 363.0976 |
| Kaempferol 3-glucuronide | 31.251 | 461.0719 | 461.07255 | 1.40 | [M-H]- | C21H18O12 | 461.0729, 462.0762, 463.0796 |
| Lamalbid | 15.678 | 423.1459 | 423.1497023 | 8.98 | [M+H]+ | C17H26O12 | 423.1459, 424.1492, 425.1526 |
| Licochalcone B | 21.751 | 285.07632 | 285.0768475 | 1.85 | [M-H]- | C16H14O5 | 285.0763, 286.0796, 287.0830 |
| Lobaric Acid | 19.547 | 455.17639 | 455.1711419 | 11.53 | [M-H]- | C25H28O8 | 455.1763,  456.1797, 457.1831 |
| Loganic acid | 17.424 | 375.12891 | 375.129671 | 2.02 | [M-H]- | C16H24O10 | 375.1289, 376.1322, 377.1356 |
| L-Saccharopine | 26.362 | 277.14011 | 277.1394124 | 2.51 | [M+H]+ | C11H20N2O6 | 277.1401, 278.1434, 279.1468 |
| Lusitanicoside | 25.385 | 443.18726 | 443.1911732 | 8.82 | [M+H]+ | C21H30O10 | 443.1872, 444.1906, 445.1939 |
| Luteolin | 25.432 | 285.03979 | 285.040462 | 2.35 | [M-H]- | C15H10O6 | 285.0398, 286.0432, 287.0466 |
| Maltitol | 4.796 | 343.12341 | 343.1245856 | 3.42 | [M-H]- | C12H24O11 | 343.1234, 344.1267, 345.1301 |
| Maltotriose | 5.093 | 503.16022 | 503.1617589 | 3.05 | [M-H]- | C18H32O16 | 503.1602, 504.1635, 505.1669 |
| Mangiferin | 22.404 | 423.08792 | 423.0921874 | 10.08 | [M+H]+ | C19H18O11 | 423.0879, 424.0912, 425.0946 |
| Mannose 6-phosphate | 24.983 | 259.02402 | 259.022443 | 6.08 | [M-H]- | C6H13O9P | 259.0240, 260.0273, 261.0307 |
| Melezitose | 5.093 | 503.16022 | 503.1617589 | 3.05 | [M-H]- | C18H32O16 | 503.1602, 504.1635, 505.1669 |
| Methoxsalen (Oxsoralen) | 24.333 | 217.04663 | 217.0495347 | 13.38 | [M+H]+ | C12H8O4 | 217.0466, 218.0499, 219.0533 |
| Methyl asterrate | 9.352 | 361.08969 | 361.0928915 | 8.86 | [M-H]- | C18H18O8 | 361.0896, 362.0930, 363.0964 |
| Mitomycin c | 16.433 | 333.11835 | 333.1204437 | 6.28 | [M-H]- | C15H18N4O5 | 333.1183, 334.1217, 335.1250 |
| Mycophenolic Acid | 24.111 | 319.11804 | 319.1187124 | 2.10 | [M-H]- | C17H20O6 | 319.1180, 320.1213, 321.1247 |
| Myricetin 3,7,3',4'-tetramethyl ether | 26.787 | 375.10571 | 375.1074435 | 4.62 | [M+H]+ | C19H18O8 | 375.1057, 376.1090, 377.1124 |
| Myricetin-3-rutinoside | 15.76 | 627.15625 | 627.1555755 | 1.07 | [M+H]+ | C27H30O17 | 627.1561, 628.1595, 629.1629 |
| 2,5-dihydroxy-3-undecylcyclohexa-2,5-diene-1,4-dione | 27.277 | 293.17856 | 293.1758333 | 9.30 | [M-H]- | C17H26O4 | 293.1785, 294.1819, 295.1852 |
| 4-methoxy-7-methylfuro[3,2-g]chromen-5-one | 4.189 | 231.06218 | 231.0651848 | 13.00 | [M+H]+ | C13H10O4 | 231.0621, 232.0655, 233.0688 |
| 2-(3,4-dihydroxyphenyl)-5,7-dihydroxy-2,3-dihydrochromen-4-one | 23.361 | 289.07001 | 289.0706641 | 2.26 | [M+H]+ | C15H12O6 | 289.0700, 290.0733, 291.0767 |
| 5,9,10-trihydroxy-2,2-dimethyl-12-(2-methylbut-3-en-2-yl)pyrano[3,2-b]xanthen-6-one | 16.6 | 393.13992 | 393.1343624 | 14.13 | [M-H]- | C23H22O6 | 393.1399, 394.1432, 395.1466 |
| Perfamine | 19.676 | 314.13763 | 314.1386841 | 3.35 | [M+H]+ | C18H19NO4 | 314.1376, 315.1409, 316.1443 |
| (2R,3S,4S,5R,6S)-2-(hydroxymethyl)-6-(3,4,5-trimethoxyphenoxy)oxane-3,4,5-triol | 20.734 | 347.13019 | 347.1336583 | 9.99 | [M+H]+ | C15H22O9 | 347.1301, 348.1335, 349.1369, |
| [(2R,3S,4R,5R,6S)-6-[(2S,3R,4S,5S,6R)-2-[5,8-dihydroxy-2-(4-methoxyphenyl)-4-oxochromen-7-yl]oxy-4,5-dihydroxy-6-(hydroxymethyl)oxan-3-yl]oxy-3,4,5-trihydroxyoxan-2-yl]methyl acetate | 20.213 | 667.18182 | 667.1868756 | 7.57 | [M+H]+ | C30H34O17 | 667.1820, 668.1853, 669.1887 |
| [(2R,3S,4R,5R,6R)-6-[2-(3,4-dihydroxyphenyl)ethoxy]-4,5-dihydroxy-2-(hydroxymethyl)oxan-3-yl] (E)-3-(3,4-dihydroxyphenyl)prop-2-enoate | 21.19 | 479.15274 | 479.1547876 | 4.27 | [M+H]+ | C23H26O11 | 479.1527, 480.1560, 481.1594 |
| 2-(3,4-dihydroxyphenyl)-5,7-dihydroxy-6-[3,4,5-trihydroxy-6-(hydroxymethyl)oxan-2-yl]chromen-4-one | 21.751 | 447.09186 | 447.0932855 | 3.18 | [M-H]- | C21H20O11 | 447.0918, 448.0952, 449.0985 |
| (2S,3S,4S,5R,6S)-3,4,5-trihydroxy-6-(5-hydroxy-6-methoxy-4-oxo-2-phenylchromen-7-yl)oxyoxane-2-carboxylic acid | 23.219 | 459.0932 | 459.0932855 | 0.18 | [M-H]- | C22H20O11 | 459.0932, 460.0965, 461.0999 |
| 2-(3,4-dihydroxyphenyl)-5-hydroxy-3,6,7-trimethoxychromen-4-one | 25.918 | 361.09091 | 361.0917935 | 2.44 | [M+H]+ | C18H16O8 | 361.0903, 362.0937, 363.0970 |
| [(2S,3R,4R,5S,6S)-2-[2-(3,4-dihydroxyphenyl)-5,7-dihydroxy-4-oxochromen-3-yl]oxy-3,5-dihydroxy-6-methyloxan-4-yl] 3,4,5-trihydroxybenzoate | 20.085 | 599.10321 | 599.1042441 | 1.72 | [M-H]- | C28H24O15 | 599.1032, 600.1065, 601.1099 |
| (3R,5R)-3,5-bis[[(E)-3-(3,4-dihydroxyphenyl)prop-2-enoyl]oxy]-1,4-dihydroxycyclohexane-1-carboxylic acid | 22.185 | 515.11847 | 515.1195002 | 1.99 | [M-H]- | C25H24O12 | 515.1184, 516.1218, 517.1251 |
| 5,7-dihydroxy-2-(4-hydroxyphenyl)-3-[(2S,3R,4S,5S,6R)-3,4,5-trihydroxy-6-[[(2R,3R,4R,5R,6S)-3,4,5-trihydroxy-6-methyloxan-2-yl]oxymethyl]oxan-2-yl]oxychromen-4-one | 20.706 | 593.14954 | 593.1511943 | 2.78 | [M-H]- | C27H30O15 | 593.1495, 594.1528, 595.1562 |
| (E)-3-[4-methoxy-2-[(2S,3R,4S,5S,6R)-3,4,5-trihydroxy-6-(hydroxymethyl)oxan-2-yl]oxyphenyl]prop-2-enoic acid | 22.056 | 374.14288 | 374.1445552 | 4.47 | [M+NH4]+ | C16H20O9 | 374.1428, 375.1462, 376.1495 |
| 1,5-dihydroxy-2,3-dimethoxy-10-methylacridin-9-one | 24.51 | 302.1015 | 302.1022986 | 2.64 | [M+H]+ | C16H15NO5 | 302.1015, 303.1048, 304.1082, |
| 5-hydroxy-2,2-dimethyl-7,10-bis(2-methylbut-3-en-2-yl)pyrano[3,2-g]chromen-8-one | 26.223 | 379.19617 | 379.1914834 | 12.35 | [M-H]- | C24H28O4 | 379.1961, 380.1995, 381.2028 |
| (E)-1-(2-hydroxy-3,4,5,6-tetramethoxyphenyl)-3-phenylprop-2-en-1-one | 4.796 | 343.12341 | 343.1187124 | 13.69 | [M-H]- | C19H20O6 | 343.1234, 344.1267, 345.1301 |
| 2-(2,6-dimethoxyphenyl)-5,6-dimethoxychromen-4-one | 24.069 | 343.11426 | 343.1176143 | 9.77 | [M+H]+ | C19H18O6 | 343.1142, 344.1176, 345.1209, |
| 6-[(2S,3R,4S,5S,6R)-4,5-dihydroxy-6-(hydroxymethyl)-3-[(2S,3R,4S,5S)-3,4,5-trihydroxyoxan-2-yl]oxyoxan-2-yl]-5,7-dihydroxy-2-(4-hydroxyphenyl)chromen-4-one | 22.403 | 565.15283 | 565.1551816 | 4.16 | [M+H]+ | C26H28O14 | 565.1528, 566.1561, 567.1595 |
| 7-[(2,6-dihydroxy-2,5,5,8a-tetramethyl-3,4,4a,6,7,8-hexahydro-1H-naphthalen-1-yl)methoxy]chromen-2-one | 20.745 | 418.26309 | 418.2587971 | 10.26 | [M+NH4]+ | C24H32O5 | 418.2630, 419.2664, 420.2698 |
| 7-hydroxy-2-(4-hydroxy-3,5-dimethoxyphenyl)-5-[(2S,3R,4S,5S,6R)-3,4,5-trihydroxy-6-(hydroxymethyl)oxan-2-yl]oxychromen-4-one | 24.685 | 493.13013 | 493.1340522 | 7.95 | [M+H]+ | C23H24O12 | 493.1301, 494.1334, 495.1368 |
| [(2S,3R,4S,5S,6R)-2-[[(3S,4R,4aR)-4-ethenyl-4a-hydroxy-8-oxo-3,4,5,6-tetrahydropyrano[3,4-c]pyran-3-yl]oxy]-4,5-dihydroxy-6-(hydroxymethyl)oxan-3-yl] 2,4-dihydroxy-6-(3-hydroxyphenyl)benzoate | 22.098 | 601.15503 | 601.1562796 | 2.07 | [M-H]- | C29H30O14 | 601.1550, 602.1583, 603.1617 |
| (2S,3S,4S,5R,6S)-6-[2-(3,4-dihydroxyphenyl)-5-hydroxy-4-oxochromen-7-yl]oxy-3,4,5-trihydroxyoxane-2-carboxylic acid | 24.64 | 461.07205 | 461.07255 | 1.08 | [M-H]- | C21H18O12 | 461.0721, 462.0754, 463.0788 |
| 8-hydroxy-6-methoxy-7-[(2S,3R,4S,5S,6R)-3,4,5-trihydroxy-6-(hydroxymethyl)oxan-2-yl]oxychromen-2-one | 5.271 | 371.09955 | 371.0972728 | 6.13 | [M+H]+ | C16H18O10 | 371.0995, 372.1029, 373.1062 |
| Nicotianamine | 4.325 | 302.13562 | 302.1357594 | 0.46 | [M-H]- | C12H21N3O6 | 302.1356, 303.1389, 304.1423 |
| Oleic acid | 5.264 | 283.26245 | 283.2631563 | 2.49 | [M+H]+ | C18H34O2 | 283.2624, 284.2658, 285.2691 |
| Ononin | 5.122 | 429.12466 | 429.1191063 | 12.94 | [M-H]- | C22H22O9 | 429.1246, 30.1280, 431.1313 |
| Orientin | 31.137 | 447.09268 | 447.0932855 | 1.35 | [M-H]- | C21H20O11 | 447.0926, 448.0960, 449.0993 |
| Oroxindin | 23.219 | 459.0932 | 459.0932855 | 0.18 | [M-H]- | C22H20O11 | 459.0932, 460.0965, 461.0999 |
| Osajin | 21.052 | 403.15948 | 403.1550979 | 10.86 | [M-H]- | C25H24O5 | 403.1594, 404.1628, 405.1661 |
| Palatinose | 4.916 | 341.10818 | 341.1089355 | 2.21 | [M-H]- | C12H22O11 | 341.1081, 342.1115, 343.1148 |
| Phlorhizin | 22.185 | 435.12836 | 435.129671 | 3.01 | [M-H]- | C21H24O10 | 435.1283, 436.1317, 437.1350 |
| Prednisolone acetate | 22.83 | 403.20752 | 403.2115147 | 9.90 | [M+H]+ | C23H30O6 | 403.2075, 404.2108, 405.2142 |
| Prostaglandin E1 | 28.527 | 353.23254 | 353.2333482 | 2.28 | [M-H]- | C20H34O5 | 353.2325, 354.2358, 355.2392 |
| Pseudo-anisatin | 23.492 | 297.13382 | 297.1343624 | 1.82 | [M-H]- | C15H22O6 | 297.1338, 298.1371, 299.1405 |
| Quercetin-3-O-pentosyl(1-2) acetylpentoside | 20.352 | 607.1275 | 607.1304588 | 4.87 | [M-H]- | C27H28O16 | 607.1275, 608.1308, 609.1342 |
| Rosmarinic acid | 25.961 | 359.07657 | 359.0772415 | 1.86 | [M-H]- | C18H16O8 | 359.0765, 360.0799, 361.0832 |
| Rotenone | 16.6 | 393.13992 | 393.1343624 | 14.13 | [M-H]- | C23H22O6 | 393.1399, 394.1432, 395.1466 |
| Rubiadin | 24.64 | 253.04997 | 253.0506328 | 2.61 | [M-H]- | C15H10O4 | 253.0499, 254.0533, 255.0566 |
| Rutin | 21.315 | 609.14172 | 609.1461089 | 7.20 | [M-H]- | C27H30O16 | 609.1417, 610.1450, 611.1484 |
| Samidin | 16.703 | 385.1344 | 385.129277 | 13.30 | [M-H]- | C21H22O7 | 385.1344, 386.1377, 387.1411 |
| Santin | 27.262 | 343.08118 | 343.0823269 | 3.34 | [M-H]- | C18H16O7 | 343.0811, 344.0845, 345.0878 |
| Sclareol | 30.013 | 331.25961 | 331.2607484 | 3.43 | [M+Na]+ | C20H36O2 | 331.2596, 332.2629, 333.2663 |
| Scutellarioside II | 21.915 | 507.14966 | 507.1508003 | 2.24 | [M-H]- | C24H28O12 | 507.1496, 508.1530, 509.1563 |
| Sinapyl alcohol | 15.849 | 211.09358 | 211.0964849 | 13.76 | [M+H]+ | C11H14O4 | 211.0935, 212.0969, 213.1002 |
| Skullcapflavone II | 26.743 | 373.09207 | 373.0928915 | 2.20 | [M-H]- | C19H18O8 | 373.0920, 374.0954, 375.0987 |
| Sternbin | 25.873 | 301.07135 | 301.0717622 | 1.36 | [M-H]- | C16H14O6 | 301.0708, 302.0741, 303.0775 |
| Sucrose | 5.224 | 341.10818 | 341.1089355 | 2.21 | [M-H]- | C12H22O11 | 341.1081, 342.1115, 343.1148 |
| Syringetin-3-O-galactoside | 15.865 | 509.12961 | 509.1289668 | 1.26 | [M+H]+ | C23H24O13 | 509.1296, 510.1329, 511.1363 |
| Syringin | 23.056 | 371.13364 | 371.1347563 | 3.00 | [M-H]- | C17H24O9 | 371.1336, 372.1369, 373.1403 |
| Tiliroside | 23.537 | 595.14276 | 595.1446169 | 3.12 | [M+H]+ | C30H26O13 | 595.1427, 596.1461, 597.1494 |
| Trehalose | 5.224 | 341.10818 | 341.1089355 | 2.21 | [M-H]- | C12H22O11 | 341.1081, 342.1115, 343.1148 |
| Tryptophan | 19.236 | 205.09685 | 205.0971536 | 1.48 | [M+H]+ | C11H12N2O2 | 205.0968, 206.1002, 207.1035 |
| Uridine | 26.092 | 245.07745 | 245.0768121 | 2.60 | [M+H]+ | C9H12N2O6 | 245.0774, 246.0808, 247.0841 |
| Ursolic acid | 29.584 | 455.35245 | 455.3530694 | 1.36 | [M-H]- | C30H48O3 | 455.3524, 456.3558, 457.3591 |
| Vedelianin | 21.272 | 481.26001 | 481.2584649 | 3.21 | [M+H]+ | C29H36O6 | 481.2600, 482.2633, 483.2667 |
| Vitexin | 22.534 | 431.0972 | 431.0983708 | 2.71 | [M-H]- | C21H20O10 | 431.0972, 432.1005, 433.1039 |
| Zeatin | 18.257 | 220.11752 | 220.1192861 | 8.02 | [M+H]+ | C10H13N5O | 220.1175, 221.1208, 222.1242 |

**Table S3** List of metabolites used for molecular docking studies

| Compound name | CID |
| --- | --- |
| (-)-Epicatechin | 72276 |
| (1R,3R,4S,5R)-1,3,4-trihydroxy-5-[(E)-3-(4-hydroxy-3-methoxyphenyl)prop-2-enoyl]oxycyclohexane-1-carboxylic acid | 10133609 |
| (2S,3R,4S,5S,6R)-2-[3-hydroxy-5-[(Z)-2-(4-hydroxyphenyl)ethenyl]phenoxy]-6-(hydroxymethyl)oxane-3,4,5-triol | 10178463 |
| (2S,3S)-2-(3,4,5-trihydroxyphenyl)-3,4-dihydro-2H-chromene-3,5,7-triol | 10425234 |
| (2S,3S)-3,5,7-trihydroxy-2-(4-hydroxyphenyl)-2,3-dihydrochromen-4-one | 9838882 |
| (E)-3-[4-[(2S,3R,4S,5S,6R)-3,4,5-trihydroxy-6-(hydroxymethyl)oxan-2-yl]oxyphenyl]prop-2-enoic acid | 9840292 |
| (Z)-3-[4-methoxy-2-[(2S,3R,4S,5S,6R)-3,4,5-trihydroxy-6-(hydroxymethyl)oxan-2-yl]oxyphenyl]prop-2-enoic acid | 24094157 |
| [6]-Gingerol | 442793 |
| 13-HODE | 5282947 |
| 2-(3,4-dimethoxyphenyl)-5,7-dihydroxy-3,6-dimethoxy-4H-chromen-4-one | 5379563 |
| 2-(3-hydroxy-5-methoxyphenoxy)-6-(hydroxymethyl)oxane-3,4,5-triol | 56776291 |
| 2',5'-Dihydroxy-4-Methoxychalcone | 5355888 |
| 2-[(2S,4aR,8aS)-2-hydroxy-4a-methyl-8-methylidene-3,4,5,6,7,8a-hexahydro-1H-naphthalen-2-yl]prop-2-enoic acid | 23757244 |
| 2-methyl-3-[(2S,3R,4S,5S,6R)-3,4,5-trihydroxy-6-(hydroxymethyl)oxan-2-yl]oxypyran-4-one | 5316639 |
| 3-p-Coumaroylquinic acid | 9945785 |
| 4-(3,4-dihydroxyphenyl)-6,7-dihydroxynaphthalene-2-carboxylic acid | 195328 |
| 4-Methylabyssinone V | 6548074 |
| 5,10-dimethoxy-2,2-dimethylpyrano[3,2-g]chromen-8-one | 155148 |
| 5,7-dihydroxy-2-(4-hydroxyphenyl)-3,6-dimethoxy-4H-chromen-4-one | 5352032 |
| 5,7-dihydroxy-2-(4-hydroxyphenyl)-6-[3,4,5-trihydroxy-6-(hydroxymethyl)oxan-2-yl]chromen-4-one | 4475102 |
| 5-[(2R,3S)-6-hydroxy-2-(4-hydroxyphenyl)-4-[(E)-2-(4-hydroxyphenyl)ethenyl]-2,3-dihydro-1-benzofuran-3-yl]benzene-1,3-diol | 5315234 |
| 5-hydroxy-3,6,7-trimethoxy-2-(4-methoxyphenyl)-4H-chromen-4-one | 5318355 |
| 7-[(E)-3-methyl-4-(4-methyl-5-oxo-2H-furan-2-yl)but-2-enoxy]chromen-2-one | 5776876 |
| 9-hydroxy-1,4a-dimethyl-7-propan-2-yl-2,3,4,9,10,10a-hexahydrophenanthrene-1-carboxylic acid | 10448477 |
| 9-hydroxy-10,12-octadecadienoic acid | 5282945 |
| 9-KODE | 9839084 |
| 9-Methoxy-2,2-dimethyl-2,6-dihydro-pyrano[3,2-c]quinolin-5-one | 648601 |
| Abietic acid | 10569 |
| Albiflorin | 24868421 |
| Aloenin | 162305 |
| Arbutin | 440936 |
| Asiatic Acid | 119034 |
| Aucubin | 91458 |
| Bakuchiol | 5468522 |
| Bicuculline (+) | 10237 |
| Cafestol | 108052 |
| Casticin | 5315263 |
| Catechin | 9064 |
| Chlorogenic acid | 1794427 |
| Coniferin | 5280372 |
| Deacetoxy-7-Oxogedunin | 6708525 |
| Dehydroabietic acid | 94391 |
| Dehydrorotenone | 303993 |
| Duguetine | 45102748 |
| Ellagic acid | 5281855 |
| Epigallocatechin | 72277 |
| Eriodictyol 7,3'-dimethyl ether | 14235076 |
| Eriodictyol | 440735 |
| Esculin | 5281417 |
| Eudesmin | 325601 |
| Feruloyltyramine | 5280537 |
| Fisetin | 5281614 |
| Formononetin | 5280378 |
| Gamma-mangostin | 5464078 |
| Geniposidic acid | 443354 |
| Ginkgolide B | 65243 |
| Goniothalenol | 442513 |
| Goyazensolide | 5358393 |
| Grayanotoxin I | 9548612 |
| Hederagenin | 73299 |
| Heralenol | 328236 |
| Irigenin | 5464170 |
| Isorhapontin | 5281716 |
| Isosakuranetin | 160481 |
| Isotaxiresinol | 9841162 |
| Isovitexin | 162350 |
| Jaceidin | 5464461 |
| Licochalcone B | 5318999 |
| Lobaric Acid | 73157 |
| loganic acid | 89640 |
| Lusitanicoside | 442799 |
| Luteolin | 5280445 |
| Myricetin 3,7,3',4'-tetramethyl ether | 5748580 |
| 2,5-dihydroxy-3-undecylcyclohexa-2,5-diene-1,4-dione | 3218 |
| 4-methoxy-7-methylfuro[3,2-g]chromen-5-one | 6716 |
| 2-(3,4-dihydroxyphenyl)-5,7-dihydroxy-2,3-dihydrochromen-4-one | 11095 |
| 5,9,10-trihydroxy-2,2-dimethyl-12-(2-methylbut-3-en-2-yl)pyrano[3,2-b]xanthen-6-one | 5281646 |
| (2R,3S,4S,5R,6S)-2-(hydroxymethyl)-6-(3,4,5-trimethoxyphenoxy)oxane-3,4,5-triol | 636454 |
| (2S,3S,4S,5R,6S)-3,4,5-trihydroxy-6-(5-hydroxy-6-methoxy-4-oxo-2-phenylchromen-7-yl)oxyoxane-2-carboxylic acid | 14655552 |
| 2-(3,4-dihydroxyphenyl)-5-hydroxy-3,6,7-trimethoxychromen-4-one | 5280699 |
| (E)-3-[4-methoxy-2-[(2S,3R,4S,5S,6R)-3,4,5-trihydroxy-6-(hydroxymethyl)oxan-2-yl]oxyphenyl]prop-2-enoic acid | 23872107 |
| 1,5-dihydroxy-2,3-dimethoxy-10-methylacridin-9-one | 14463129 |
| 5-hydroxy-2,2-dimethyl-7,10-bis(2-methylbut-3-en-2-yl)pyrano[3,2-g]chromen-8-one | 5315945 |
| (E)-1-(2-hydroxy-3,4,5,6-tetramethoxyphenyl)-3-phenylprop-2-en-1-one | 6442392 |
| 2-(2,6-dimethoxyphenyl)-5,6-dimethoxychromen-4-one | 629965 |
| 7-[(2,6-dihydroxy-2,5,5,8a-tetramethyl-3,4,4a,6,7,8-hexahydro-1H-naphthalen-1-yl)methoxy]chromen-2-one | 495442 |
| 8-hydroxy-6-methoxy-7-[(2S,3R,4S,5S,6R)-3,4,5-trihydroxy-6-(hydroxymethyl)oxan-2-yl]oxychromen-2-one | 11508953 |
| Nicotianamine | 9882882 |
| Ononin | 442813 |
| Oroxindin | 3084961 |
| Osajin | 95168 |
| Phlorhizin | 6072 |
| Pseudo-anisatin | 6708809 |
| Rosmarinic acid | 5281792 |
| Rotenone | 6758 |
| Rubiadin | 124062 |
| Sclareol | 163263 |
| Sinapyl alcohol | 5280507 |
| Skullcapflavone II | 124211 |
| Syringin | 5316860 |
| Ursolic Acid | 64945 |
| Vedelianin | 643539 |
| Vitexin | 5280441 |

**Table S4** Molecular docking of co-crystallized ligand with hub targets

| **SL No.** | **Co-crystallized ligand name** | **Docking score** |
| --- | --- | --- |
| **Hub target: ESR1 (PDB ID: 6VPF)** | | |
| 1 | Clomifene | -11.234 |
| 2 | Clomifene | -8.218 |
| **Hub target: AKT1 (PDB ID: 8UW7)** | | |
| 3 | 4-{2-[({4-[(2P)-2-(2-aminopyridin-3-yl)-5-phenyl-3H-imidazo[4,5-b]pyridin-3-yl]phenyl}methyl)amino]ethyl}-2-hydroxybenzaldehyde | -11.681 |
| 4 | 4-{2-[({4-[(2P)-2-(2-aminopyridin-3-yl)-5-phenyl-3H-imidazo[4,5-b]pyridin-3-yl]phenyl}methyl)amino]ethyl}-2-hydroxybenzaldehyde | -9.617 |
| 5 | 4-{2-[({4-[(2P)-2-(2-aminopyridin-3-yl)-5-phenyl-3H-imidazo[4,5-b]pyridin-3-yl]phenyl}methyl)amino]ethyl}-2-hydroxybenzaldehyde | -8.427 |
| **Hub target STAT3 (PDB ID: 6NJS)** | | |
| 1 | [(2-{[(5S,8S,10aR)-3-acetyl-8-({(2S)-5-amino-1-[(diphenylmethyl)amino]-1,5-dioxopentan-2-yl}carbamoyl)-6-oxodecahydropyrrolo[1,2-a][1,5]diazocin-5-yl]carbamoyl}-1H-indol-5-yl)(difluoro)methyl]phosphonic acid | -10.1 |
| 2 | [(2-{[(5S,8S,10aR)-3-acetyl-8-({(2S)-5-amino-1-[(diphenylmethyl)amino]-1,5-dioxopentan-2-yl}carbamoyl)-6-oxodecahydropyrrolo[1,2-a][1,5]diazocin-5-yl]carbamoyl}-1H-indol-5-yl)(difluoro)methyl]phosphonic acid | -9.917 |
| 3 | [(2-{[(5S,8S,10aR)-3-acetyl-8-({(2S)-5-amino-1-[(diphenylmethyl)amino]-1,5-dioxopentan-2-yl}carbamoyl)-6-oxodecahydropyrrolo[1,2-a][1,5]diazocin-5-yl]carbamoyl}-1H-indol-5-yl)(difluoro)methyl]phosphonic acid | -9.316 |
| 4 | [(2-{[(5S,8S,10aR)-3-acetyl-8-({(2S)-5-amino-1-[(diphenylmethyl)amino]-1,5-dioxopentan-2-yl}carbamoyl)-6-oxodecahydropyrrolo[1,2-a][1,5]diazocin-5-yl]carbamoyl}-1H-indol-5-yl)(difluoro)methyl]phosphonic acid | -9.16 |
| 5 | [(2-{[(5S,8S,10aR)-3-acetyl-8-({(2S)-5-amino-1-[(diphenylmethyl)amino]-1,5-dioxopentan-2-yl}carbamoyl)-6-oxodecahydropyrrolo[1,2-a][1,5]diazocin-5-yl]carbamoyl}-1H-indol-5-yl)(difluoro)methyl]phosphonic acid | -8.446 |
| 6 | [(2-{[(5S,8S,10aR)-3-acetyl-8-({(2S)-5-amino-1-[(diphenylmethyl)amino]-1,5-dioxopentan-2-yl}carbamoyl)-6-oxodecahydropyrrolo[1,2-a][1,5]diazocin-5-yl]carbamoyl}-1H-indol-5-yl)(difluoro)methyl]phosphonic acid | -8.141 |
| 7 | [(2-{[(5S,8S,10aR)-3-acetyl-8-({(2S)-5-amino-1-[(diphenylmethyl)amino]-1,5-dioxopentan-2-yl}carbamoyl)-6-oxodecahydropyrrolo[1,2-a][1,5]diazocin-5-yl]carbamoyl}-1H-indol-5-yl)(difluoro)methyl]phosphonic acid | -8.014 |
| 8 | [(2-{[(5S,8S,10aR)-3-acetyl-8-({(2S)-5-amino-1-[(diphenylmethyl)amino]-1,5-dioxopentan-2-yl}carbamoyl)-6-oxodecahydropyrrolo[1,2-a][1,5]diazocin-5-yl]carbamoyl}-1H-indol-5-yl)(difluoro)methyl]phosphonic acid | -7.988 |
| 9 | [(2-{[(5S,8S,10aR)-3-acetyl-8-({(2S)-5-amino-1-[(diphenylmethyl)amino]-1,5-dioxopentan-2-yl}carbamoyl)-6-oxodecahydropyrrolo[1,2-a][1,5]diazocin-5-yl]carbamoyl}-1H-indol-5-yl)(difluoro)methyl]phosphonic acid | -7.854 |
| 10 | [(2-{[(5S,8S,10aR)-3-acetyl-8-({(2S)-5-amino-1-[(diphenylmethyl)amino]-1,5-dioxopentan-2-yl}carbamoyl)-6-oxodecahydropyrrolo[1,2-a][1,5]diazocin-5-yl]carbamoyl}-1H-indol-5-yl)(difluoro)methyl]phosphonic acid | -7.606 |
| 11 | [(2-{[(5S,8S,10aR)-3-acetyl-8-({(2S)-5-amino-1-[(diphenylmethyl)amino]-1,5-dioxopentan-2-yl}carbamoyl)-6-oxodecahydropyrrolo[1,2-a][1,5]diazocin-5-yl]carbamoyl}-1H-indol-5-yl)(difluoro)methyl]phosphonic acid | -7.485 |
| 12 | [(2-{[(5S,8S,10aR)-3-acetyl-8-({(2S)-5-amino-1-[(diphenylmethyl)amino]-1,5-dioxopentan-2-yl}carbamoyl)-6-oxodecahydropyrrolo[1,2-a][1,5]diazocin-5-yl]carbamoyl}-1H-indol-5-yl)(difluoro)methyl]phosphonic acid | -7.474 |
| 13 | [(2-{[(5S,8S,10aR)-3-acetyl-8-({(2S)-5-amino-1-[(diphenylmethyl)amino]-1,5-dioxopentan-2-yl}carbamoyl)-6-oxodecahydropyrrolo[1,2-a][1,5]diazocin-5-yl]carbamoyl}-1H-indol-5-yl)(difluoro)methyl]phosphonic acid | -7.298 |
| 14 | [(2-{[(5S,8S,10aR)-3-acetyl-8-({(2S)-5-amino-1-[(diphenylmethyl)amino]-1,5-dioxopentan-2-yl}carbamoyl)-6-oxodecahydropyrrolo[1,2-a][1,5]diazocin-5-yl]carbamoyl}-1H-indol-5-yl)(difluoro)methyl]phosphonic acid | -7.053 |
| 15 | [(2-{[(5S,8S,10aR)-3-acetyl-8-({(2S)-5-amino-1-[(diphenylmethyl)amino]-1,5-dioxopentan-2-yl}carbamoyl)-6-oxodecahydropyrrolo[1,2-a][1,5]diazocin-5-yl]carbamoyl}-1H-indol-5-yl)(difluoro)methyl]phosphonic acid | -7.033 |
| 16 | [(2-{[(5S,8S,10aR)-3-acetyl-8-({(2S)-5-amino-1-[(diphenylmethyl)amino]-1,5-dioxopentan-2-yl}carbamoyl)-6-oxodecahydropyrrolo[1,2-a][1,5]diazocin-5-yl]carbamoyl}-1H-indol-5-yl)(difluoro)methyl]phosphonic acid | -6.957 |
| 17 | [(2-{[(5S,8S,10aR)-3-acetyl-8-({(2S)-5-amino-1-[(diphenylmethyl)amino]-1,5-dioxopentan-2-yl}carbamoyl)-6-oxodecahydropyrrolo[1,2-a][1,5]diazocin-5-yl]carbamoyl}-1H-indol-5-yl)(difluoro)methyl]phosphonic acid | -6.937 |
| 18 | [(2-{[(5S,8S,10aR)-3-acetyl-8-({(2S)-5-amino-1-[(diphenylmethyl)amino]-1,5-dioxopentan-2-yl}carbamoyl)-6-oxodecahydropyrrolo[1,2-a][1,5]diazocin-5-yl]carbamoyl}-1H-indol-5-yl)(difluoro)methyl]phosphonic acid | -6.707 |
| 19 | [(2-{[(5S,8S,10aR)-3-acetyl-8-({(2S)-5-amino-1-[(diphenylmethyl)amino]-1,5-dioxopentan-2-yl}carbamoyl)-6-oxodecahydropyrrolo[1,2-a][1,5]diazocin-5-yl]carbamoyl}-1H-indol-5-yl)(difluoro)methyl]phosphonic acid | -6.458 |
| 20 | [(2-{[(5S,8S,10aR)-3-acetyl-8-({(2S)-5-amino-1-[(diphenylmethyl)amino]-1,5-dioxopentan-2-yl}carbamoyl)-6-oxodecahydropyrrolo[1,2-a][1,5]diazocin-5-yl]carbamoyl}-1H-indol-5-yl)(difluoro)methyl]phosphonic acid | -6.26 |
| 21 | [(2-{[(5S,8S,10aR)-3-acetyl-8-({(2S)-5-amino-1-[(diphenylmethyl)amino]-1,5-dioxopentan-2-yl}carbamoyl)-6-oxodecahydropyrrolo[1,2-a][1,5]diazocin-5-yl]carbamoyl}-1H-indol-5-yl)(difluoro)methyl]phosphonic acid | -6.167 |
| 22 | [(2-{[(5S,8S,10aR)-3-acetyl-8-({(2S)-5-amino-1-[(diphenylmethyl)amino]-1,5-dioxopentan-2-yl}carbamoyl)-6-oxodecahydropyrrolo[1,2-a][1,5]diazocin-5-yl]carbamoyl}-1H-indol-5-yl)(difluoro)methyl]phosphonic acid | -5.536 |
| 23 | [(2-{[(5S,8S,10aR)-3-acetyl-8-({(2S)-5-amino-1-[(diphenylmethyl)amino]-1,5-dioxopentan-2-yl}carbamoyl)-6-oxodecahydropyrrolo[1,2-a][1,5]diazocin-5-yl]carbamoyl}-1H-indol-5-yl)(difluoro)methyl]phosphonic acid | -5.429 |
| 24 | [(2-{[(5S,8S,10aR)-3-acetyl-8-({(2S)-5-amino-1-[(diphenylmethyl)amino]-1,5-dioxopentan-2-yl}carbamoyl)-6-oxodecahydropyrrolo[1,2-a][1,5]diazocin-5-yl]carbamoyl}-1H-indol-5-yl)(difluoro)methyl]phosphonic acid | -5.388 |
| 25 | [(2-{[(5S,8S,10aR)-3-acetyl-8-({(2S)-5-amino-1-[(diphenylmethyl)amino]-1,5-dioxopentan-2-yl}carbamoyl)-6-oxodecahydropyrrolo[1,2-a][1,5]diazocin-5-yl]carbamoyl}-1H-indol-5-yl)(difluoro)methyl]phosphonic acid | -5.14 |
| 26 | [(2-{[(5S,8S,10aR)-3-acetyl-8-({(2S)-5-amino-1-[(diphenylmethyl)amino]-1,5-dioxopentan-2-yl}carbamoyl)-6-oxodecahydropyrrolo[1,2-a][1,5]diazocin-5-yl]carbamoyl}-1H-indol-5-yl)(difluoro)methyl]phosphonic acid | -5.049 |
| 27 | [(2-{[(5S,8S,10aR)-3-acetyl-8-({(2S)-5-amino-1-[(diphenylmethyl)amino]-1,5-dioxopentan-2-yl}carbamoyl)-6-oxodecahydropyrrolo[1,2-a][1,5]diazocin-5-yl]carbamoyl}-1H-indol-5-yl)(difluoro)methyl]phosphonic acid | -4.973 |
| 28 | [(2-{[(5S,8S,10aR)-3-acetyl-8-({(2S)-5-amino-1-[(diphenylmethyl)amino]-1,5-dioxopentan-2-yl}carbamoyl)-6-oxodecahydropyrrolo[1,2-a][1,5]diazocin-5-yl]carbamoyl}-1H-indol-5-yl)(difluoro)methyl]phosphonic acid | -4.474 |
| 29 | [(2-{[(5S,8S,10aR)-3-acetyl-8-({(2S)-5-amino-1-[(diphenylmethyl)amino]-1,5-dioxopentan-2-yl}carbamoyl)-6-oxodecahydropyrrolo[1,2-a][1,5]diazocin-5-yl]carbamoyl}-1H-indol-5-yl)(difluoro)methyl]phosphonic acid | -3.996 |
| 30 | [(2-{[(5S,8S,10aR)-3-acetyl-8-({(2S)-5-amino-1-[(diphenylmethyl)amino]-1,5-dioxopentan-2-yl}carbamoyl)-6-oxodecahydropyrrolo[1,2-a][1,5]diazocin-5-yl]carbamoyl}-1H-indol-5-yl)(difluoro)methyl]phosphonic acid | -3.964 |
| 31 | [(2-{[(5S,8S,10aR)-3-acetyl-8-({(2S)-5-amino-1-[(diphenylmethyl)amino]-1,5-dioxopentan-2-yl}carbamoyl)-6-oxodecahydropyrrolo[1,2-a][1,5]diazocin-5-yl]carbamoyl}-1H-indol-5-yl)(difluoro)methyl]phosphonic acid | -3.905 |
| 32 | [(2-{[(5S,8S,10aR)-3-acetyl-8-({(2S)-5-amino-1-[(diphenylmethyl)amino]-1,5-dioxopentan-2-yl}carbamoyl)-6-oxodecahydropyrrolo[1,2-a][1,5]diazocin-5-yl]carbamoyl}-1H-indol-5-yl)(difluoro)methyl]phosphonic acid | -3.428 |
| **Hub target: ERBB2 (PDB ID: 2A91)** | | |
| 1 | 2-acetamido-2-deoxy-beta-D-glucopyranose | -6.892 |
| 2 | 2-acetamido-2-deoxy-beta-D-glucopyranose | -6.731 |
| 3 | 2-acetamido-2-deoxy-beta-D-glucopyranose | -6.722 |
| 4 | 2-acetamido-2-deoxy-beta-D-glucopyranose | -6.423 |
| 5 | 2-acetamido-2-deoxy-beta-D-glucopyranose | -6.37 |
| 6 | 2-acetamido-2-deoxy-beta-D-glucopyranose | -6.174 |
| 7 | 2-acetamido-2-deoxy-beta-D-glucopyranose | -6.134 |
| 8 | 2-acetamido-2-deoxy-beta-D-glucopyranose | -6.01 |
| 9 | 2-acetamido-2-deoxy-beta-D-glucopyranose | -5.9 |
| 10 | 2-acetamido-2-deoxy-beta-D-glucopyranose | -5.828 |
| 11 | 2-acetamido-2-deoxy-beta-D-glucopyranose | -5.816 |
| 12 | 2-acetamido-2-deoxy-beta-D-glucopyranose | -5.717 |
| 13 | 2-acetamido-2-deoxy-beta-D-glucopyranose | -5.648 |
| 14 | 2-acetamido-2-deoxy-beta-D-glucopyranose | -5.588 |
| 15 | 2-acetamido-2-deoxy-beta-D-glucopyranose | -5.411 |
| 16 | 2-acetamido-2-deoxy-beta-D-glucopyranose | -5.364 |
| **Hub target: BCL2 (PDB ID: 8HTS)** | | |
| 1 | 4-[2-[(2~{S})-2-(2-cyclopropylphenyl)pyrrolidin-1-yl]-7-azaspiro[3.5]nonan-7-yl]-~{N}-[3-nitro-4-(oxan-4-ylmethylamino)phenyl]sulfonyl-2-(1~{H}-pyrrolo[2,3-b]pyridin-5-yloxy)benzamide | -4.309 |
| 2 | 4-[2-[(2~{S})-2-(2-cyclopropylphenyl)pyrrolidin-1-yl]-7-azaspiro[3.5]nonan-7-yl]-~{N}-[3-nitro-4-(oxan-4-ylmethylamino)phenyl]sulfonyl-2-(1~{H}-pyrrolo[2,3-b]pyridin-5-yloxy)benzamide | -1.26 |
| 3 | 4-[2-[(2~{S})-2-(2-cyclopropylphenyl)pyrrolidin-1-yl]-7-azaspiro[3.5]nonan-7-yl]-~{N}-[3-nitro-4-(oxan-4-ylmethylamino)phenyl]sulfonyl-2-(1~{H}-pyrrolo[2,3-b]pyridin-5-yloxy)benzamide | 1.309 |
| **Hub target: EGFR (PDB ID: 3POZ)** | | |
| 1 | N-{2-[4-({3-chloro-4-[3-(trifluoromethyl)phenoxy]phenyl}amino)-5H-pyrrolo[3,2-d]pyrimidin-5-yl]ethyl}-3-hydroxy-3-methylbutanamide | -4.542 |
| **Hub target: IL6 (PDB ID: 1ALU)** | | |
| 1 | L(+)-Tartaric acid | -4.151 |
| 2 | L(+)-Tartaric acid | -4.097 |
| 3 | L(+)-Tartaric acid | -3.898 |
